# Basophil activation test discriminates between allergy and tolerance in peanut-sensitized children

**DOI:** 10.1016/j.jaci.2014.04.039

**Published:** 2014-09

**Authors:** Alexandra F. Santos, Abdel Douiri, Natalia Bécares, Shih-Ying Wu, Alick Stephens, Suzana Radulovic, Susan M.H. Chan, Adam T. Fox, George Du Toit, Victor Turcanu, Gideon Lack

**Affiliations:** a Division of Asthma, Allergy & Lung Biology, Department of Pediatric Allergy, King's College London, London, United Kingdom; b MRC & Asthma UK Centre in Allergic Mechanisms of Asthma, London, United Kingdom; c Immunoallergology Department, Coimbra University Hospital, Coimbra, Portugal; d Gulbenkian Programme for Advanced Medical Education, Lisbon, Portugal; e Department of Public Health Science, School of Medicine, King's College London, London, United Kingdom; f National Institute for Health Research, Biomedical Research Centre, Guy's and St Thomas' Hospital NHS Foundation Trust, London, United Kingdom

**Keywords:** Anaphylaxis, basophil activation test, CD203c, CD63, diagnosis, flow cytometry, food allergy, peanut allergy, ROC curve, BAT, Basophil activation test, CRD, Component-resolved diagnosis, DBPCFC, Double-blind placebo-controlled food challenge, fMLP, Formyl-methionyl-leucyl-phenylalanine, NA, Non–peanut-sensitized nonallergic, OFC, Oral food challenge, PA, Peanut-allergic, PPV, Positive predictive value, PS, Peanut-sensitized but tolerant, P-sIgE, Peanut-specific IgE, ROC, Receiver-operating characteristic, sIgE, Specific IgE, SPT, Skin prick test

## Abstract

**Background:**

Most of the peanut-sensitized children do not have clinical peanut allergy. In equivocal cases, oral food challenges (OFCs) are required. However, OFCs are laborious and not without risk; thus, a test that could accurately diagnose peanut allergy and reduce the need for OFCs is desirable.

**Objective:**

To assess the performance of basophil activation test (BAT) as a diagnostic marker for peanut allergy.

**Methods:**

Peanut-allergic (n = 43), peanut-sensitized but tolerant (n = 36) and non–peanut-sensitized nonallergic (n = 25) children underwent skin prick test (SPT) and specific IgE (sIgE) to peanut and its components. BAT was performed using flow cytometry, and its diagnostic performance was evaluated in relation to allergy versus tolerance to peanut and validated in an independent population (n = 65).

**Results:**

BAT in peanut-allergic children showed a peanut dose-dependent upregulation of CD63 and CD203c while there was no significant response to peanut in peanut-sensitized but tolerant (*P* < .001) and non–peanut-sensitized nonallergic children (*P* < .001). BAT optimal diagnostic cutoffs showed 97% accuracy, 95% positive predictive value, and 98% negative predictive value. BAT allowed reducing the number of required OFCs by two-thirds. BAT proved particularly useful in cases in which specialists could not accurately diagnose peanut allergy with SPT and sIgE to peanut and to Arah2. Using a 2-step diagnostic approach in which BAT was performed only after equivocal SPT or Arah2-sIgE, BAT had a major effect (97% reduction) on the number of OFCs required.

**Conclusions:**

BAT proved to be superior to other diagnostic tests in discriminating between peanut allergy and tolerance, particularly in difficult cases, and reduced the need for OFCs.

Ten percent of North American children are sensitized to peanut,^1^ but only 1.4% are clinically allergic to peanut.^2^ The gold standard for the diagnosis of peanut allergy is double-blind placebo-controlled food challenge (DBPCFC); however, this is time-consuming and carries the risk of causing an acute allergic reaction.^3^ Therefore, in clinical practice, whenever possible, the diagnosis of peanut allergy is based on the combination of a history of an immediate-type allergic reaction to peanut together with *in vivo* or in *vitro* measurement of sensitization.^4^ Some clinics use peanut-specific IgE (P-sIgE) alone, others use peanut skin prick test (SPT) alone, and some such as ours use a combination of these tests. No clear consensus exists as to which is the best approach. The diagnosis of peanut allergy can be particularly difficult in cases in which there is no clear history of peanut consumption. With increasing awareness about food allergy and the fact that many families avoid peanut in the first few years of life, peanut-sensitized children with no history of oral exposure to peanut constitute a considerable proportion of patients seen in allergy clinics. This has resulted in a marked increase in the number of oral food challenge (OFC) requests. Thus, a test that could accurately diagnose peanut allergy reducing the need for OFC is desirable and would change clinical practice.

To try to improve the utility of SPT and P-sIgE, diagnostic decision values have been determined.^5^, ^6^, ^7^, ^8^, ^9^ However, a large proportion of peanut-sensitized children have SPT and P-sIgE results below these cutoffs, falling in the immunologic gray area^10^ (see Fig E1 in this article's Online Repository at www.jacionline.org). Ara h 2 is a dominant allergen and has been proved to be particularly useful for diagnosis^11^, ^12^; however, peanut allergy can develop in patients with undetectable specific IgE (sIgE) levels to Ara h 2 and other major peanut allergens.^12^, ^13^, ^14^

The basophil activation test (BAT) to peanut is an *in vitro* assay in which the expression of activation markers on the surface of basophils is evaluated by using flow cytometry after stimulation with peanut allergens.^15^, ^16^ It can be performed using 1 mL of blood without requiring cell separation. We sought to assess the performance of BAT in the diagnosis of peanut allergy and to compare it with existing diagnostic tests.

## Methods

### Study population

Peanut-allergic (PA), peanut-sensitized but tolerant (PS), and non–peanut-sensitized nonallergic (NA) children were prospectively and consecutively enrolled from our Pediatric Allergy service on the days when the investigator (A.F.S.) was available to perform BAT. The allergic status to peanut was determined by using OFCs, except for (1) children with a convincing history of systemic reaction(s) to peanut within 1 year of their visit and (a) wheal size of SPT of 8 mm or more^8^ and/or (b) P-sIgE level of 15 KU_A_/L or more,^8^ who were considered peanut allergic; and (2) children (15 NA and 5 PS) who were able to eat 4 g or more of peanut protein twice a week (as assessed by a validated peanut consumption questionnaire^17^) without developing allergic symptoms, who were considered peanut tolerant. Peanut sensitization was defined by a wheal size of SPT of 1 mm or more and/or P-sIgE level of 0.10 KU_A_/L or more.

All children underwent clinical evaluation, SPT, P-sIgE determination, component-resolved diagnosis (CRD), and OFC, as appropriate. An additional sample of blood was drawn in lithium heparin (BD Vacutainer, Plymouth, United Kingdom) for BAT, which was performed within 4 hours of blood collection. The study was approved by the South East London Research Ethics Committee 2, and written informed consent was obtained from parents of all children.

### Skin prick testing, serum-sIgE, and OFCs

SPT was performed using peanut extract (ALK-Abelló, Hørsholm, Denmark), as previously described.^18^ The level of sIgE (peanut and CRD) was measured using an immunoenzymatic assay (ImmunoCAP, ThermoFisher, Uppsala, Sweden).

DBPCFC consisted of 6 verum doses and 3 placebo doses randomly interspersed with verum doses up to a cumulative dose of 9.35 g of peanut protein (see Table E1 in this article's Online Repository at www.jacionline.org). Children of 1 to 3 years were given 1 placebo and 5 verum doses up to a cumulative dose of 4.35 g of peanut protein. In infants (≤1 year), the OFCs were open up to a cumulative dose of 4.35 g of peanut protein. Nine older children also received an open OFC for logistical reasons. OFCs were considered negative when all doses were tolerated. If an allergic reaction developed at any stage after a verum dose, the OFC was considered positive (see Table E2 in this article's Online Repository at www.jacionline.org) and the symptoms treated. If a reaction followed a placebo dose, the patient was brought in for 2-day challenge (1 day placebo and 1 day verum).^19^

### Basophil activation test

Heparinized whole blood was stimulated for 30 minutes at 37°C with peanut extract (ALK Abelló) diluted in RPMI medium (GIBCO, Paisley, United Kingdom) at serial 10-fold dilutions from 10 μg/mL to 0.1 ng/mL. For details about the extract and allergen concentrations, see this article's Online Repository at www.jacionline.org.^20^ Polyclonal goat antihuman IgE (1 μg/mL, Sigma-Aldrich, Poole, United Kingdom), monoclonal mouse antihuman FcɛRI (2.5 μg/mL, eBioscience, San Diego, Calif), formyl-methionyl-leucyl-phenylalanine (fMLP, 1 μM, Sigma-Aldrich), or RPMI medium alone were used as controls. Before erythrocyte lysis, cells were stained with CD123-FITC (eBioscience), CD203c-PE, HLA-DR-PerCP, and CD63-APC (Biolegend, San Diego, Calif). Basophils were gated as SSC^low^/CD203c+/CD123+/HLA-DR− (see Fig E2 in this article's Online Repository at www.jacionline.org). Basophil expression of CD63 and CD203c was evaluated using FACS CantoII with FACSDiva software (BD Biosciences, San Jose, Calif). The flow cytometry data were analyzed using FlowJo software (version 7.6.1; TreeStar, Ashland, Ore) by an investigator who was blinded to the clinical features of the participants. Basophil activation was expressed as %CD63^+^ basophils and as the stimulation index of the mean fluorescence intensity (MFI) of CD203c.

### Statistical analysis

We estimated that a sample of 32 PA and 32 PS children would give us 99% power, at a 2-sided type I error probability of 0.05, to detect a significant difference in the %CD63^+^ basophils after peanut stimulation between PA and PS on the basis of data from a previous study.^21^

Qualitative variables were compared between PA and PS children using the Fisher exact test or χ^2^ tests, and continuous variables were compared using the Mann-Whitney *U* test or the Kruskal-Wallis test.

The performance of allergy tests was examined against the allergic status to peanut using receiver-operating characteristic (ROC)-curve analyses. The cutoffs to predict peanut allergy and peanut tolerance for BAT and the various allergy tests with optimal accuracy were determined and validated. We performed internal validation using repeated random subsampling validation (bootstrap) and “leave-one-out” methodologies.^22^ Both methodologies produced similar results in estimating the optimal cutoff points, and the former methodology is reported. The 95% CI was constructed using bootstrapping methodology with 1000 replications to reflect on the reproducibility.^23^ An external validation study was also conducted using a new cohort of 65 subjects (25 PA, 24 PS, and 16 NA) mainly recruited from the Peanut Allergy Sensitization study, a group of patients from all over the country who were excluded from the Learning Early About Peanut Allergy study,^18^ and from a private Pediatric Allergy clinic in London. The cutoffs previously determined in the primary study population were applied to this validation study population and sensitivity, specificity, predictive values, likelihood ratios, and accuracy were calculated.

Three Pediatric Allergy specialist attending physicians were asked to classify 44 equivocal cases from the primary study population as peanut allergic or tolerant on the basis of history and results of SPT, P-sIgE, and CRD. The agreement between physicians was calculated as percentages and assessed with κ statistics.^24^

Statistical analyses were performed with SPSS 20.0 and STATA 12.1 for Windows. Significance was determined using a 2-sided α level of 0.05.

### Combination of BAT with other diagnostic tests

In the primary study population, after ROC-curve analyses, we compared the performance of BAT with SPT, P-sIgE, and Arah2-sIgE using conventional cutoffs.^6^, ^8^, ^12^ We further assessed the diagnostic utility of BAT when considered in combination with other allergy tests, that is, considering the results of different tests simultaneously, and when considered as a second or third step in the diagnostic process, that is, performed in selected patients in whom the results of single or of combinations of tests were equivocal.

When interpreted individually, the results of standard allergy tests were considered diagnostic of allergy when the positive predictive value cutoff was 95% or more, diagnostic of tolerance when the negative predictive value (NPV) cutoff was less than 95%, and equivocal when between the positive and the negative cutoffs (Fig E1). For BAT, we used the cutoff for the mean of %CD63^+^ basophils at 10 and 100 ng/mL of peanut extract and considered BAT equivocal in the case of “nonresponders” (defined as <5% CD63^+^ basophils to IgE-mediated controls and ≥5% CD63%^+^ basophils to fMLP).

The combination of allergy tests was interpreted as equivocal if one test result was 95% or more PPV cutoff and another test result was less than 95% NPV cutoff or when all tests gave equivocal results (as defined above) or a combination of equivocal results and results less than 95% NPV.

In these simulations, OFCs were deemed required when the interpretation of tests was equivocal. The combination of SPT and P-sIgE was the clinical reference point against which the change in the number of OFCs required was determined.

## Results

### Study participants

One hundred nine children, 76% boys, aged from 5 months to 17 years (median, 5 years), participated in the study (see Fig E3 in this article's Online Repository at www.jacionline.org). Sixty-six OFCs to peanut were performed: 20 positive, 41 negative, and 5 indeterminate (3 patients refused to eat and 2 showed subjective symptoms in the absence of objective signs). These 5 patients were excluded. Among the study participants (n = 104, 43 PA and 61 peanut-tolerant), 48 patients underwent DBPCFC and 13 open OFCs. Demographic and clinical features of the study population are represented in Table I and in Figs E4 and E5 in this article's Online Repository at www.jacionline.org.

**Table I:** Demographic and clinical features of the whole primary study population (n = 104) and of the subgroup of the primary study population with equivocal clinical history and inconclusive SPT and sIgE results (n = 44)

Characteristic	Primary study population (n = 104)				Subpopulation with equivocal allergy test results (n = 44)		
	PA (n = 43)	Peanut tolerant (n = 61)		*P* value∗	PA (n = 8)	PS (n = 36)	*P* value
		PS (n = 36)	NA (n = 25)				
Age (y)	5.5 (1.5-17.0)	4.0 (0.5-13.0)	5.0 (0.8-13.5)	.005†	5.0 (2.0-6.0)	4.0 (0.5-13.0)	.964
Males	32 (74.4)	23 (63.9)	18 (72.0)	.366	4 (50.0)	23 (63.9)	.690
History of oral exposure to peanut	26 (60.5)	7 (19.4)	15 (60.0)	<.001†	0 (0)	7 (19.5)	.618
SPT to peanut (mm)	9 (2-19)	2 (0-12)	0 (0-0)	<.001†	7 (2-9)	2 (0-12)	.002†
sIgE to peanut (KU_A_/L)	14.50 (0.14-604.0)	0.81 (0.01-35.70)	0.01 (0-0.08)	<.001†	0.94 (0.14-14.50)	0.81 (0.01-35.70)	.964
sIgE to Ara h 1 (KU_A_/L)	0.45 (0-199.0)	0.06 (0-3.79)	0.01 (0-0.03)	.001†	0.03 (0.01-8.67)	0.06 (0-3.79)	.622
sIgE to Ara h 2 (KU_A_/L)	9.21 (0.05-386.0)	0.06 (0.01-1.84)	0.01 (0-0.08)	<.001†	0.15 (0.05-8.95)	0.06 (0.01-1.84)	.023†
sIgE to Ara h 3 (KU_A_/L)	0.06 (0-89.60)	0.05 (0-1.36)	0.01 (0-0.04)	.217	0.01 (0.01-1.62)	0.05 (0-1.36)	.189
sIgE to Ara h 8 (KU_A_/L)	0.08 (0-57.80)	0.01 (0-35.80)	0.01 (0-0.02)	.027†	0.01 (0.01-4.66)	0.01 (0-35.80)	.893
sIgE to Ara h 9 (KU_A_/L)	0.01 (0-5.62)	0.02 (0-11.0)	0.01 (0-0.02)	.602	0.01 (0.01-0.28)	0.02 (0-11.0)	.823
Other food allergy	39 (90.7)	32 (88.9)	3 (12.0)	1.0	8 (100)	32 (88.9)	1.0
Atopic eczema	36 (83.7)	21 (58.3)	12 (48.0)	.022†	5 (62.5)	21 (58.3)	1.0
Asthma	13 (30.2)	6 (16.7)	0 (0)	.193	0 (0)	6 (16.7)	.573
Allergic rhinitis	14 (32.6)	9 (25.0)	2 (8.0)	.620	0 (0)	9 (25.0)	.175
Pollen allergy	14 (32.6)	8 (22.2)	1 (4.0)	.349	0 (0)	8 (22.2)	.284
Nonatopic	0 (0)	0 (0)	12 (48.0)	—	0 (0)	0 (0)	—

Values are expressed as no. (%) or median (range).

∗ *P* value refers to the comparison between PA and PS patients.

† *P* < .05.

### BAT discriminates between allergic and tolerant children

The basophils of 12 (11.5%) children were “nonresponders” and were necessarily excluded from the comparison of BAT results between groups and from the ROC-curve analysis; however, they were taken into account when assessing the clinical application of BAT and its effect in the reduction of OFCs.

**Fig 1:**
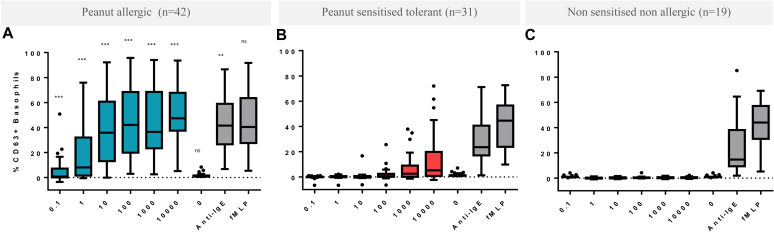
BAT to peanut in PA (n = 42, **A**), PS (n = 31, **B**), and NA (n = 19, **C**) children. The *P* value refers to the comparison of the median %CD63^+^ basophils at selected doses between PA and PS patients: ***P < .001, **P < .01, and ns, nonsignificant. 0 represents the negative control, and anti-IgE and fMLP are the positive controls.

In PA children, basophils showed increased expression of CD63 and CD203c, with increasing concentrations of peanut extract up to 100 ng/mL followed by a *plateau*. The basophils from PS children did not significantly respond to peanut (*P* < .001 for the comparison of the median basophil activation between PA and PS patients) neither did basophils from NA children (Fig 1; see Fig E6 in this article's Online Repository at www.jacionline.org). This difference in basophil response between groups was reflected in other parameters of BAT (see Table E3 in this article's Online Repository at www.jacionline.org). The %CD63^+^ basophils in response to the negative control (*P* = .958) and non–IgE-mediated positive control (fMLP, *P* = .581) was similar across groups. The proportion of nonresponders was higher in peanut-tolerant (including PS and NA) than in peanut-allergic (*P* = .012) children. Similar findings were observed for the stimulation index of CD203c.

### Diagnostic cutoff values

Peanut allergy (based on OFCs or 95% PPV, n = 42) and tolerance status (based on OFCs or consumption, n = 50) was the reference point to evaluate the diagnostic performance of BAT on ROC-curve analysis (Table E3).

The best diagnostic cutoff values (although all cutoffs performed well without statistically significant differences between them) were obtained for %CD63^+^ basophils at 100 ng/mL and mean %CD63^+^ basophils at 10 and 100 ng/mL of peanut extract (Table II). These were simultaneously optimal, negative, and positive decision levels, with 98% sensitivity, 96% specificity, 95% PPV, 98% NPV, and 97% accuracy. See Table E4 in this article's Online Repository at www.jacionline.org for optimal cutoffs for other BAT parameters.

**Table II:** Optimal cutoffs for the parameters of BAT to peanut with the largest area under the ROC curve (n = 92)

BAT parameters	Cutoff	AUC ROC	Sensitivity (%)	Specificity (%)	PPV (%)	NPV (%)	LR+	LR−	Diagnostic accuracy (%)
%CD63^+^ peanut extract 100 ng/mL	8.11 (2.93-16.47)	0.97 (0.93-1.0)	97.6 (87.4-99.9)	96.0 (86.3-99.5)	95.3 (84.2-99.4)	98.0 (89.1-99.9)	24.4 (6.3-95.0)	0.02 (0.0-0.17)	96.7 (93.1-100)
SI CD203c peanut extract 100 ng/mL	1.88 (1.62-2.24)	0.96 (0.91-1.0)	95.2 (83.8-99.4)	96.0 (86.3-99.5)	95.2 (83.8-99.4)	96.0 (86.3-99.5)	23.8 (6.1-92.7)	0.05 (0.01-0.19)	95.7 (91.5-99.8)
Mean %CD63^+^ peanut extract 10-100 ng/mL	4.78 (4.78-11.76)	0.97 (0.93-1.0)	97.6 (87.4-99.9)	96.0 (86.3-99.5)	95.3 (84.2-99.4)	98.0 (89.1-99.9)	24.4 (6.3-95.0)	0.02 (0.0-0.17)	96.7 (93.1-100)
Mean SI CD203c peanut extract 10-100 ng/mL	1.40 (1.40-1.75)	0.97 (0.94-1.0)	100.0 (91.6-100)	94.0 (83.5-98.7)	93.3 (81.7-98.6)	100.0 (92.5-100)	16.7 (5.6-49.9)	—∗	96.7 (93.1-100)

Values in parentheses represent 95% CI.

*AUC*, Area under the ROC curve; *LR+*, positive likelihood ratio; *LR−*, negative likelihood ratio; *MFI*, mean fluorescence intensity; *%CD63*^*+*^, percentage of CD63-positive basophils (corrected for the negative control); *SI CD203c*, stimulation index of CD203c (MFI CD203c poststimulation/MFI CD203c of negative control).

∗ LR− could not be determined because sensitivity was 100%.

**Fig 2:**
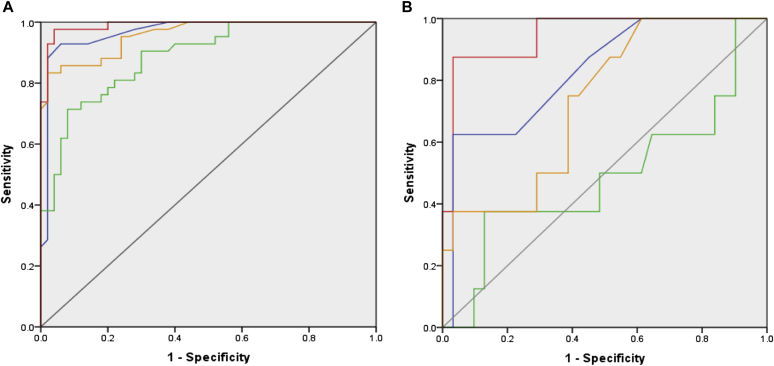
ROC curves for BAT *(red)*, SPT *(blue)*, P-sIgE *(green)*, and sIgE to Ara h 2 *(orange)* for the whole study population (n = 92) **(A)** and children with equivocal history, SPT, and sIgE to peanut and its components (n = 39) **(B)**. For BAT, the average between %CD63^+^ basophils at 10 and 100 ng/mL of peanut extract was considered. For area under the ROC curves for different tests in the 2 study populations, see Table E5.

The area under the ROC curve for BAT was superior to that for other allergy tests (Fig 2, *A*; see Table E5 in this article's Online Repository at www.jacionline.org). Arah2-sIgE performed better than did sIgE to other peanut components (see Fig E7 in this article's Online Repository at www.jacionline.org).

### Validation of diagnostic cutoff values in an independent population

To externally validate our findings, we prospectively recruited 65 children (25 PA, 24 PS, and 16 NA; see Table E6 in this article's Online Repository at www.jacionline.org) who underwent the same study procedures as the primary study population. The vast majority (94%) underwent OFCs, and all positive OFCs were DBPCFC. Applying the optimal cutoff previously determined for the mean of %CD63^+^ basophils at 10 and 100 ng/mL of peanut extract, BAT showed 100% specificity, 83.3% sensitivity, 100% PPV, 90.2% NPV, and 93.4% accuracy and was superior to SPT, sIgE, and Arah2-sIgE (see Table E7 in this article's Online Repository at www.jacionline.org).

### The use of BAT in peanut-sensitized children with equivocal diagnosis

The utility of BAT was further assessed in the subgroup (n = 44) of the primary study population with equivocal history and inconclusive results of SPT, P-sIgE, and CRD (Table I and Fig 2, *B*).

Three Pediatric Allergy specialist attending physicians were asked to classify them as peanut allergic or tolerant on the basis of available information. In most of the cases (46% to 64%), the physicians could not decide without doing an OFC. They correctly diagnosed 26% to 36% and misclassified 9% to 16% of the cases (14% false negatives). Agreement between the 3 pairs of physicians was poor to fair, with κ values of 0.16, 0.29, and 0.36. The 3 specialists agreed in 16 (36%) cases: 4 correctly diagnosed, 1 misclassified, and in 11 cases they were unable to decide. In contrast, BAT provided 36 (82%) correct diagnoses, 2 (5%) false positives, and 1 (2%) false negative and required 5 (11%) OFCs. Excluding nonresponders, BAT had a diagnostic accuracy of 95% (Table E5).

### The combination of BAT with other diagnostic tests

We evaluated the diagnostic performance of different tests in the primary study population (n = 104), including BAT nonresponders, in 3 ways (Table III): considering each test on its own; considering the results of different diagnostic tests simultaneously; and considering BAT as a second or third sequential step in the diagnostic process, performed in patients in whom the results of single or combinations of standard allergy tests were equivocal.

**Table III:** Performance of allergy tests in the diagnosis of peanut allergy (N = 104)

Single diagnostic test	Correct diagnoses∗	No. of false positives	No. of false negatives	No. of BATs	No. of OFCs	Change in the no. of OFC†
SPT	78 (75)	1 (1)	1 (1)	—	24 (23)	−12 (−33)
P-sIgE	57 (55)	3 (3)	3 (3)	—	41 (39)	+5 (+13)
Ara h 2	82 (79)	1 (1)	2 (2)	—	19 (18)	−17 (−46)
BAT‡	89 (86§)	2 (2)	1 (1)	104 (100)	12 (12)	−24 (−67)

Combination of diagnostic tests	Correct diagnoses∗	No. of false positives	No. of false negatives	No. of BATs	No. of OFCs	Change in the no. of OFCs†
*SPT + P-sIgE*	*67 (64)*	*1 (1)*	*0 (0)*	*—*	*36 (35)*	*0 (0)*
P-sIgE + Ara h 2	66 (63)	1 (1)	1 (1)	—	36 (35)	0 (0)
P-sIgE + BAT	66 (63)	1 (1)	2 (2)	104 (100)	35 (34)	−1 (−3)
SPT + BAT	77 (74)	2 (2)	0 (0)	104 (100)	25 (25)	−11 (−31)
SPT + Ara h 2	78 (75)	1 (1)	0 (0)	—	25 (24)	−11 (−31)
Ara h 2 + BAT	77 (74)	1 (1)	2 (2)	104 (100)	24 (24)	−12 (−33)
SPT + P-sIgE + Ara h 2	67 (64)	1 (1)	0 (0)	—	36 (35)	0 (0)
SPT + Ara h 2 + BAT	70 (67)	1 (1)	0 (0)	104 (100)	33 (33)	−3 (−8)
SPT + P-sIgE + BAT	63 (61)	2 (2)	0 (0)	104 (100)	39 (38)	+3 (+8)
P-sIgE + Ara h 2 + BAT	63 (61)	1 (1)	1 (1)	104 (100)	39 (38)	+3 (+8)
SPT + P-sIgE + Ara h 2 + BAT	60 (58)	1 (1)	0 (0)	104 (100)	43 (42)	+7 (+19)

BAT as a second step in the diagnostic process	Correct diagnoses∗	No. of false positives	No. of false negatives	No. of BATs	No. of OFCs	Change in the no. of OFCs†
SPT → BAT	98 (94)	3 (3)	2 (2)	24 (23)	1 (1)	−35 (−97)
P-sIgE → BAT	93 (89)	5 (5)	3 (3)	41 (39)	3 (3)	−33 (−92)
Ara h 2 → BAT	99 (95)	2 (2)	2 (2)	19 (18)	1 (1)	−35 (−97)
SPT + P-sIgE → BAT	97 (93)	3 (3)	1 (1)	36 (35)	3 (3)	−33 (−92)
SPT + Ara h 2 → BAT	99 (95)	2 (2)	1 (1)	38 (37)	2 (2)	−34 (−94)
SPT + P-sIgE + Ara h 2 → BAT	96 (92)	3 (3)	1 (1)	36 (35)	4 (4)	−32 (−89)

BAT as a third step in the diagnostic process	Correct diagnoses∗	No. of false positives	No. of false negatives	No. of BATs	No. of OFCs	Change in the no. of OFCs†
SPT → Ara h 2 → BAT	98 (94)	3 (3)	3 (3)	6 (6)	0 (0)	−36 (−100)

Results are presented as number of patients (% of total study population).

*Ara h 2*, sIgE to Ara h 2.

∗ The proportion of correct diagnoses was determined as (“true-positives” + “true-negatives”)/104.

† Reduction in the number of OFCs was calculated in comparison with the number of OFCs after SPT and sIgE (ie, 36 OFCs, row in *italic*); negative numbers represent a decrease and positive numbers an increase in the number of OFCs required.

‡ For BAT, we used 4.78% for the average of CD63^+^ basophils at 10 and 100 ng/mL of peanut extract as the diagnostic cutoff point.

§ For BAT, excluding nonresponders, the proportion of correct diagnoses is 96.7%.

Considering single tests, BAT performed best and allowed a reduction in the number of OFCs by two-thirds, followed by Arah2-sIgE and SPT. P-sIgE on its own performed the poorest, conferring the highest number of OFCs and correctly diagnosing only 55% of the patients. Considering combinations of allergy tests, it was best to combine 2 different tests as opposed to 3 or 4 tests. All combinations of tests required an increase between 2- and 3.5-fold in the number of OFCs compared with BAT alone.

With a view to apply BAT in clinical practice, we assessed the role of BAT as a second or third step in the diagnostic workup, which would require a smaller number of BATs (Table III; see Fig E8 in this article's Online Repository at www.jacionline.org). The 2-step strategy significantly reduced the number of OFCs, more than using Arah2-sIgE as a second step to SPT or to P-sIgE (see Table E8 in this article's Online Repository at www.jacionline.org), as proposed by Dang et al.^12^ The 3-step sequential strategy of SPT→Arah2-sIgE→BAT (Table III and Fig E8) further reduced the number of OFCs to zero at the expense of a slightly higher number of false-negative results (n = 3).

## Discussion

To arrive at a correct diagnosis of peanut allergy or tolerance, a considerable proportion of peanut-sensitized patients seen in allergy clinics need to undergo an OFC. Specialized centers have become overwhelmed with the increasing number of OFC requests, and overdiagnosis of peanut allergy due to overreliance on allergy tests alone is common. There is a large immunological gray area between 95% PPV and 95% NPV cutoffs for SPT, P-sIgE, and Arah2-sIgE (Fig E1). If we apply a single cutoff value based on the ROC-curve point-of-inflexion, the diagnostic accuracy of these tests suffers. In BAT, the ROC-curve optimal cutoff acted simultaneously as positive and negative cutoff with no immunologic gray area, allowing for a significant reduction in the number of OFCs, even among difficult patients with conflicting history and results of SPT, P-sIgE, and CRD. Unlike for these tests, for BAT, we were able to use the ROC-curve point-of-inflexion as a single cutoff value while maintaining a 97% diagnostic accuracy.

Our study is the largest study assessing the role of BAT in the diagnosis of peanut allergy.^21^, ^25^, ^26^ It is the first study to prospectively validate BAT in an independent population and to evaluate its diagnostic performance on its own, in combination and sequentially with other allergy tests, as well as its effect on the number of OFCs. We studied a large population, including not only sensitized but also nonsensitized nonallergic patients. Although peanut-induced basophil activation would not be expected in the absence of P-sIgE, it was important to demonstrate the specificity of BAT in NA patients. BAT maintained its good performance in an independent population prospectively recruited to validate the diagnostic cutoffs. In 44 children with evidence of sensitization and conflicting allergy test results, 3 specialist doctors showed poor agreement and were unable to decide in most of the cases whether they were peanut allergic without doing an OFC, while BAT still performed very well in this subgroup.

One of the strengths of our study is that participants were carefully clinically phenotyped, the vast majority by OFCs. In the primary study population, 23 patients were assumed to have peanut allergy on the basis of SPT and/or P-sIgE of 95% or more PPV cutoffs (previously validated in our patient population^8^) and positive history. This is a potential weakness of the study; however, given the extremely high probability that such patients would react clinically, we decided on clinical and ethical grounds not to challenge them. Most of the patients who were challenged underwent DBPCFC (48 of 61), but 4 children 1 year or younger and another 9 older children underwent open OFCs. This is a limitation of our study. However, most (7 of 9) of the older children undergoing open OFCs had negative challenges (open OFC is the gold standard for peanut tolerance) and the 2 who had a positive OFC had objective unequivocal signs of an allergic reaction immediately after peanut ingestion, consistent with the new Practall guidelines' criteria for a positive OFC.^27^ In 5 patients (4.6%), the OFCs were inconclusive, which highlights the fact that although DBPCFC is the gold standard, it is not foolproof in the diagnosis of peanut allergy.^28^ BAT may prove particularly useful in cases in which OFC cannot be performed or is indeterminate. In the external validation population, 94% of the patients were challenged and all positive OFCs were DBPCFC.

The main limitation of BAT was the patients with nonresponder basophils, rendering BAT uninterpretable. The proportion of nonresponders we found (11.5% in the primary study population and 6.2% in the external validation population) was similar to that previously described.^21^, ^29^, ^30^, ^31^ This is analogous, for example, to situations in which SPT cannot be interpreted because of a negative histamine control or in which P-sIgE cannot be interpreted in the light of a high polyclonal IgE production or indeed when an OFC is inconclusive. Importantly, these are not misdiagnosed patients but cases in which BAT is uninterpretable and the diagnostic workup needs to be taken further, namely, by doing an OFC. The fact that nonresponders were almost exclusively (92%) peanut-tolerant patients raises the question whether basophil unresponsiveness through the IgE-mediated pathway could be a mechanism underlying peanut tolerance. Another limitation was that different peanut extracts were used for different tests; however, all extracts contained the major peanut allergens. Furthermore, our study was performed in children recruited in a specialized clinical setting and thus may not reflect the results of BAT to peanut in adults or the general population. Further limitations to consider when applying BAT in clinical practice are the fact that BAT needs to be performed on live cells, soon after blood collection, and requires flow cytometry equipment and appropriately trained staff.

Following the evaluation of the diagnostic performance of each test by ROC-curve analysis, we wanted to assess their effect on the reduction of OFCs. The effect of BAT was different in the 3 scenarios considered: single tests, combination of tests, and BAT as a sequential step in the diagnostic process (Table III). Very few studies have addressed the utility of combinations of allergy tests, and this deficiency has been highlighted as an unmet clinical need in the National Institute of Allergy and Infectious Diseases–sponsored food allergy guidelines.^32^ Considering single tests, BAT performed best, followed closely by Ara h2-sIgE and SPT, even when patients with nonresponder basophils were taken into account. P-sIgE performed the poorest and conferred the highest number of OFCs. Surprisingly, the different combinations of tests provided little, if any, advantage compared with BAT alone, with a uniform reduction in the percentage of correct diagnoses and a significant increase in the number of OFCs required. Disappointingly, the combination of tests did not result in a consistent decrease in the number of false-negative outcomes. Performing BAT as a sequential step reduced the number of BATs required (Table III and Fig E6) and had a major effect in reducing the number of OFCs regardless of the test performed as first line. For instance, performing BAT after SPT or after Arah2-sIgE allowed a 97% reduction in the number of OFCs compared with the combination of SPT and P-sIgE (our routine clinical reference point) and a 92% reduction compared with BAT alone. However, this was at the expense of 2 or 3 false-negative outcomes. To prevent any false-negative cases from occurring using this sequential test approach, we would need to challenge all the BAT-negative patients in addition to the patients with equivocal BAT; even in this more conservative scenario, the total number of OFCs was significantly reduced by 64% (SPT→BAT) or 69% (Arah2-sIgE→BAT) compared with combining SPT and P-sIgE. The decision on whether to increase the number of OFCs or of BATs, both reducing the possibility of false-negative tests, would depend on a cost-benefit analysis. We believe that SPT→BAT is better than Arah2-sIgE→BAT for practical reasons (SPT provides immediate results while Arah2-sIgE→BAT would require 2 separate blood collections) and given regional differences in the patterns of sensitization to peanut allergens.^13^ The 3-step diagnostic strategy further reduced the number of BATs required and eliminated the need for OFCs but this was at the expense of a higher false-negative rate, not from BATs but from SPT and Arah2-sIgE. For further discussion, see this article's Online Repository at www.jacionline.org.

To conclude, considering SPT, P-sIgE, CRD, and BAT, BAT has the best diagnostic profile. Combinations of tests offer no significant advantage to BAT alone and led to an increase in the number of OFCs. The most accurate and cost-effective analysis appears to be that of using a 2-step sequential approach in which SPT or Arah2-sIgE is followed by BAT in equivocal cases. To maximize safety and decrease false-negative tests to 0%, the 2-step sequential approach can be modified to do OFCs in the cases with equivocal BAT as well as in BAT-negative patients. We should bear in mind the limitations of OFC (3% false-negative^28^ and 2%-9% indeterminate outcomes^33^, ^34^). Future studies will determine whether BAT can add to the OFC as an *in vitro* gold standard.

**Clinical implications**

The basophil activation test to peanut can be performed in cases in which standard allergy tests have failed to diagnose peanut allergy before considering oral food challenges.

## Supplementary data

Online Repository Data

Tables E1–E8

Fig E1

Fig E2

Fig E3

Fig E4

Fig E5

Fig E6

Fig E7

Fig E8

## References

[1] Branum A.M., Lukacs S.L. (2009). Food allergy among children in the United States. Pediatrics.

[2] Sicherer S.H., Munoz-Furlong A., Godbold J.H., Sampson H.A. (2010). US prevalence of self-reported peanut, tree nut, and sesame allergy: 11-year follow-up. J Allergy Clin Immunol.

[3] Perry T.T., Matsui E.C., Conover-Walker M.K., Wood R.A. (2004). Risk of oral food challenges. J Allergy Clin Immunol.

[4] Sicherer S.H., Wood R.A. (2013). Advances in diagnosing peanut allergy. J Allergy Clin Immunol Pract.

[5] Sampson H.A., Ho D.G. (1997). Relationship between food-specific IgE concentrations and the risk of positive food challenges in children and adolescents. J Allergy Clin Immunol.

[6] Sampson H.A. (2001). Utility of food-specific IgE concentrations in predicting symptomatic food allergy. J Allergy Clin Immunol.

[7] Rance F., Abbal M., Lauwers-Cances V. (2002). Improved screening for peanut allergy by the combined use of skin prick tests and specific IgE assays. J Allergy Clin Immunol.

[8] Roberts G., Lack G. (2005). Diagnosing peanut allergy with skin prick and specific IgE testing. J Allergy Clin Immunol.

[9] van Nieuwaal N.H., Lasfar W., Meijer Y., Kentie P.A., Flinterman A.E., Pasmans S.G. (2010). Utility of peanut-specific IgE levels in predicting the outcome of double-blind, placebo-controlled food challenges. J Allergy Clin Immunol.

[10] Roberts G., Lack G. (2000). Food allergy–getting more out of your skin prick tests. Clin Exp Allergy.

[11] Nicolaou N., Murray C., Belgrave D., Poorafshar M., Simpson A., Custovic A. (2011). Quantification of specific IgE to whole peanut extract and peanut components in prediction of peanut allergy. J Allergy Clin Immunol.

[12] Dang T.D., Tang M., Choo S., Licciardi P.V., Koplin J.J., Martin P.E. (2012). Increasing the accuracy of peanut allergy diagnosis by using Ara h 2. J Allergy Clin Immunol.

[13] Vereda A., van Hage M., Ahlstedt S., Ibanez M.D., Cuesta-Herranz J., van Odijk J. (2011). Peanut allergy: clinical and immunologic differences among patients from 3 different geographic regions. J Allergy Clin Immunol.

[14] Lieberman J.A., Glaumann S., Batelson S., Borres M.P., Sampson H.A., Nilsson C. (2013). The utility of peanut components in the diagnosis of IgE-mediated peanut allergy among distinct populations. J Allergy Clin Immunol Pract.

[15] Ebo D.G., Bridts C.H., Hagendorens M.M., Aerts N.E., De Clerck L.S., Stevens W.J. (2008). Basophil activation test by flow cytometry: present and future applications in allergology. Cytometry B Clin Cytom.

[16] Chirumbolo S., Vella A., Ortolani R., De Gironcoli M., Solero P., Tridente G. (2008). Differential response of human basophil activation markers: a multi-parameter flow cytometry approach. Clin Mol Allergy.

[17] Fox A.T., Sasieni P., du Toit G., Syed H., Lack G. (2009). Household peanut consumption as a risk factor for the development of peanut allergy. J Allergy Clin Immunol.

[18] Du Toit G., Roberts G., Sayre P.H., Plaut M., Bahnson H.T., Mitchell H. (2013). Identifying infants at high risk of peanut allergy: the Learning Early About Peanut Allergy (LEAP) screening study. J Allergy Clin Immunol.

[19] Marrs T., Du Toit G., Fox A.T., Perkin M., Lack G. (2013). Double-blind food challenges can be conducted effectively by using interspersed active and placebo doses among children. J Allergy Clin Immunol.

[20] Brough H.A., Makinson K., Penagos M., Maleki S.J., Cheng H., Douiri A. (2013). Distribution of peanut protein in the home environment. J Allergy Clin Immunol.

[21] Ocmant A., Mulier S., Hanssens L., Goldman M., Casimir G., Mascart F. (2009). Basophil activation tests for the diagnosis of food allergy in children. Clin Exp Allergy.

[22] Poggio T., Rifkin R., Mukherjee S., Niyogi P. (2004). General conditions for predictivity in learning theory. Nature.

[23] Zhou X.H., Qin G. (2005). Improved confidence intervals for the sensitivity at a fixed level of specificity of a continuous-scale diagnostic test. Stat Med.

[24] Viera A.J., Garrett J.M. (2005). Understanding interobserver agreement: the kappa statistic. Fam Med.

[25] Glaumann S., Nopp A., Johansson S.G., Rudengren M., Borres M.P., Nilsson C. (2012). Basophil allergen threshold sensitivity, CD-sens, IgE-sensitization and DBPCFC in peanut-sensitized children. Allergy.

[26] Javaloyes G., Goikoetxea M.J., Garcia Nunez I., Sanz M.L., Blanca M., Scheurer S. (2012). Performance of different in vitro techniques in the molecular diagnosis of peanut allergy. J Investig Allergol Clin Immunol.

[27] Sampson H.A., Gerth van Wijk R., Bindslev-Jensen C., Sicherer S., Teuber S.S., Burks A.W. (2012). Standardizing double-blind, placebo-controlled oral food challenges: American Academy of Allergy, Asthma & Immunology-European Academy of Allergy and Clinical Immunology PRACTALL consensus report. J Allergy Clin Immunol.

[28] Caffarelli C., Petroccione T. (2001). False-negative food challenges in children with suspected food allergy. Lancet.

[29] Rubio A., Vivinus-Nebot M., Bourrier T., Saggio B., Albertini M., Bernard A. (2011). Benefit of the basophil activation test in deciding when to reintroduce cow’s milk in allergic children. Allergy.

[30] Ebo D.G., Hagendorens M.M., Bridts C.H., Schuerwegh A.J., De Clerck L.S., Stevens W.J. (2005). Flow cytometric analysis of in vitro activated basophils, specific IgE and skin tests in the diagnosis of pollen-associated food allergy. Cytometry B Clin Cytom.

[31] Ford L.S., Bloom K.A., Nowak-Wegrzyn A.H., Shreffler W.G., Masilamani M., Sampson H.A. (2013). Basophil reactivity, wheal size, and immunoglobulin levels distinguish degrees of cow’s milk tolerance. J Allergy Clin Immunol.

[32] Boyce J.A., Assa'ad A., Burks A.W., Jones S.M., Sampson H.A., Wood R.A. (2010). Guidelines for the diagnosis and management of food allergy in the United States: report of the NIAID-sponsored expert panel. J Allergy Clin Immunol.

[33] Ludman S., Ballabeni P., Eigenmann P.A., Wassenberg J. (2013). Predicting positive food challenges in children sensitised to peanuts/tree nuts. Pediatr Allergy Immunol.

[34] Nolan R.C., Richmond P., Prescott S.L., Mallon D.F., Gong G., Franzmann A.M. (2007). Skin prick testing predicts peanut challenge outcome in previously allergic or sensitized children with low serum peanut-specific IgE antibody concentration. Pediatr Allergy Immunol.

