# Supplementary material for: Basophil activation test discriminates between allergy and tolerance in peanut-sensitized children
Source: J Allergy Clin Immunol. 2014 Sep;134(3):645–52. doi: 10.1016/j.jaci.2014.04.039 (PMC4164910; doi:10.1016/j.jaci.2014.04.039)
Supplement: Online Repository Data [file mmc1.docx]

**Online Repository Material**

**E-Methods:**

**Peanut extract used in the basophil activation assay**

The peanut extract used in the basophil activation assay was prepared at ALK-Abello as previously described^1^. Briefly, raw peanut was extracted in cold isotonic phosphate buffer pH 6.5 at 100 mg/ml and magnetically stirred for 90 ± 15 minutes at 2-8°C. The extract was centrifuged at 20,000 G for 30 ± 5 minutes at 2‑8 °C then clarified by filtration through glass-fibre filters or 0.5 μm membrane filters. The pH was checked and adjusted to 6.5 ± 0.5. The extract was sterilized by filtering it twice through 0.2 μm filters then filled at 1ml per vial into sterilized glass vials and freeze dried. The frozen lyophilized extract was gradually brought to room temperature and reconstituted in PBS at 20mg/ml before use. The concentration of the major peanut allergens Ara h 1 (5.5 mg/ml), Ara h 2 (1.6 mg/ml) and Ara h 3 (4 mg/ml) in the crude extract was measured by SDS-PAGE and densitometry, Experion capillary electrophoresis and ELISA.

**E-Discussion**

Following the evaluation of the diagnostic performance of each test by ROC curve analysis, we wanted to assess the utility of the different allergy tests in the diagnosis of peanut allergy in clinical practice and their ability to reduce the need for OFC compared with our current strategy of combining SPT and specific IgE to peanut. We assessed this in three ways: considering each test on its own (Table III-"single diagnostic tests"); considering the results of different diagnostic tests simultaneously (Table III- "combination of diagnostic tests"); and considering BAT as a second or third sequential step in the diagnostic process, performed in patients where the results of single or combinations of standard allergy tests were equivocal (Table III-"BAT as a second step in the diagnostic process" and "BAT as a third step in the diagnostic process"). We considered the entire study population, including patients with non-responder basophils, and interpreted the results of the different tests by applying conventional diagnostic cut-offs^2, 3^. The results of tests when interpreted individually were considered equivocal if they fell between the negative and the positive conventional cut-offs for SPT^2^, peanut-specific IgE^2^ and Ara h 2-specific IgE^3^ or when different allergy tests gave contradictory results. The change in the number of OFC was compared with the strategy of combining SPT and specific IgE currently adopted in our clinic to decide about the need for OFC.

Considering each test alone, BAT performed best, allowing a two-third reduction in the number of OFC (Table III). The next best allergy test was Ara h 2-specific IgE followed by SPT. Specific IgE to peanut performed the poorest, correctly diagnosing only 55% of patients. BAT is a unique test as it is a functional test and does not present any "immunological grey area" for the patients for whom there is an outcome. The same cut-off (based on the ROC point of inflexion 4.78%) that allowed confirming peanut allergy with 95% certainty allowed excluding the diagnosis with 98% certainty. The positive likelihood ratio (LR+) of the elected cut-offs of BAT was 24.4 for the point of inflexion which is above the level of 10 at which a test is clinically useful^4^, resulting in a high post-test probability irrespective of the pre-test probability. Conversely, the negative likelihood ratio of BAT was extremely low, which proves very useful in excluding the diagnosis of peanut allergy given a negative test result.

When combining results of different diagnostic tests before deciding about the need for OFC, the best was to combine two tests among SPT, Ara h 2-specific IgE and BAT (Table III). Adding more tests (i.e. considering the results of 3 or 4 tests) did not significantly reduce and often increased the number of OFC because in the cases where the tests gave contradictory results, OFC were required. Combining two allergy tests compared to BAT alone decreased the false-negative cases by 2% (SPT + BAT) or decreased the false-positive cases by 1% (Ara h 2-specific IgE + BAT), depending on the specific combination. In particular, adding Ara h 2-specific IgE to SPT and specific IgE to peanut did not improve the diagnosis. Adding specific IgE to peanut to other tests resulted in no change or in an increase in the number of OFC, further putting in question the diagnostic value of specific IgE to peanut when other tests are available.

With the view of applying BAT in daily practice, we considered it as a second step in the diagnostic process, performed only in patients where the results of conventional allergy tests were equivocal (Table III), which would require a smaller number of BAT. In this sequential approach, BAT significantly reduced the number of OFC, regardless of the test or tests used as first line. For instance, performing BAT following SPT or Ara h 2-specific IgE allowed a 97% reduction in the number of OFC compared to the combination of SPT and specific IgE to peanut. This approach of preceding BAT with SPT or Ara h 2-specific IgE was also superior to using BAT alone, allowing an 83% reduction in the number of OFC. However, this resulted in 2 or 3% false-negative outcomes. In order to eliminate false-negative cases, OFC could be done not only in patients with inconclusive BAT but also in patients with negative BAT. In this case, BAT would reduce the need for OFC by 64% (SPT→BAT) to 69% (Ara h 2-specific IgE→BAT), as calculated by the sum of OFC in patients with negative and equivocal BAT in comparison with the number of OFC following combination of SPT and specific IgE to peanut. The decision to increase the number of BAT and OFC to reduce the possibility of false-negative outcomes would depend on a cost-benefit analysis.

A sequential approach was previously proposed by Dang et al^3^ to test the accuracy and practical applicability of Ara h 2-specific IgE in the diagnosis of peanut allergy. When we applied the exact same approach of considering Ara h 2-specific IgE as a second step in the diagnosis of peanut allergy in cases where SPT or specific IgE to peanut were equivocal to our own study population and compared it to using BAT, we observed that BAT provided a higher proportion of correctly diagnosed patients, a lower number of false-negative diagnosis and a greater reduction in the number of OFC, compared to Ara h 2-specific IgE (Table E7). One possible explanation for the superiority of BAT compared to Ara h 2-specific IgE is that BAT is a functional assay that takes into account the combined ability of different IgE molecules, when cross-linked by different peanut allergens present in the extract to trigger basophil degranulation, including high and low affinity IgE antibodies directed to major and minor peanut allergens (not only Ara h 2 and some of which we did not test on ImmunoCAP). Besides, Ara h 2 is not the most representative peanut allergen in some geographical locations^5^ and even in areas where it is dominant like in the United States, Australia and the United Kingdom, studies have reported cases of peanut allergy negative to all tested peanut components^3, 6, 7^. Also in this study, there was one case of a peanut allergic child with undetectable specific IgE to Ara h 2 and to the other peanut components.

In conclusion, in the absence of a clear history, SPT and specific IgE to peanut and its components are often equivocal and specialists are unable to achieve a correct diagnosis without an OFC. BAT proved superior to other diagnostic tests with an accuracy of 97%. In clinical practice, BAT could be used as a second step in the diagnostic process and performed in patients with equivocal results of SPT or Ara h 2-specific IgE before deciding on whether to do an OFC. Using a 3-step strategy eliminated the need for OFC but resulted in a high number of false-negative cases. Being a diagnostic marker for allergy versus tolerance, BAT may be useful for monitoring the natural acquisition of oral tolerance to peanut^8, 9^ and the response to immuno-modulatory treatments in patients with confirmed peanut allergy, such as allergen-specific immunotherapy^10-13^ and omalizumab^14-16^, in relation to clinical improvement.

**Legends of figures in the online repository:**

**Figure E1 -** Immunological grey area of different allergy tests: specific IgE to peanut^2, 17^, skin prick test^2^, specific IgE to Ara h 2^3^ and BAT to peanut.

Abbreviations: NA, non allergic; PA, peanut allergic; SIgE, specific IgE; SPT, skin prick test; BAT, basophil activation test; CD63^10-100^, average percentage of CD63-positive basophils at 10 and 100ng/ml of peanut extract.

**Figure E2 -** Gating strategy. Basophils were identified in whole blood in the lymphocyte-monocyte area (A) as SSC^low^ CD203c+ (B), CD123+ HLADR- (C) cells. CD63 expression was evaluated in resting (D) and activated (E) basophils as well as CD203c (F). Basophil activation by 100 ng/ml peanut extract in a peanut allergic patient is represented (E, F).

**Figure E3.** Study population (n=104).

Footnotes: *as assessed by a previously validated peanut-consumption questionnaire^17^. ^1^13 positive OFC, 4 negative OFC, 2 indeterminate OFC; ^2^6 positive OFC, 23 negative OFC, 3 equivocal OFC; ^3^10 negative OFC.

Abbreviations: SPT, skin prick test to peanut; sIgE, specific IgE to peanut; OFC, oral food challenge to peanut; posit., positive; negat., negative.

**Figure E4.** Basophil activation test to peanut in peanut allergic (PA, n=42, Fig.A), peanut-sensitized but tolerant (PS, n=31, Fig. B) and non allergic (NA, n=19, Fig. C) children. The p value refers to the comparison of the median %CD63+ basophils at selected doses between PA and PS patients: ***p<0.001, **p<0.01 and ns, non-significant. 0 represents the negative control and anti-IgE and fMLP are the positive controls.

**Figure E5 -** Indications for testing for peanut allergy in the study population: 84 children had suspected peanut allergy and 20 controls were tested only for research purposes. *This indicates the 25 children who were assigned to the total NA group we studied.

**Figure E6 -** Results of skin prick test to peanut (SPT), specific IgE to peanut (Peanut-sIgE) and specific IgE to peanut components Ara h 1, Ara h 2, Ara h 3, Ara h 8 and Ara h9, in the three patient groups: peanut allergic (PA), peanut sensitized but tolerant (PS) and non allergic (NA).

**Figure E7 -** ROC curve (area under the curve [95% CI]) for different allergy tests (n=92): basophil activation test (BAT, AUC=0.99 [0.98; 1.0]), skin prick test (SPT, AUC=0.97 [0.93; 1.0]), peanut-specific IgE (PsIgE, AUC=0.89[0.82; 0.95]), specific IgE to Arah1 (AUC=0.80 [0.71;0.89]), Arah2 (AUC=0.96[0.92; 99]), Arah3 (AUC=0.67 [0.56;0.79]), Arah8 (AUC=0.70 [0.58;0.81]) and Ara h 9 (AUC=0.56[0.44; 0.68]).

**Figure E8.** Diagnosing peanut allergy using BAT sequentially in the cases where SPT, specific IgE to peanut and/or Ara h 2-specific IgE were equivocal (equivocal cases are marked in bold). In this simulation, OFC were done in cases where BAT was indeterminate (i.e. “non-responders”). A-C: 2-step diagnostic strategy where BAT follows single tests; D-F, 2-step diagnostic strategy where BAT follows combinations of tests; G, 3-step diagnostic strategy.

Footnote: Abbreviations: SPT, skin prick test; sIgE, specific IgE; Ara h 2, specific IgE to Ara h 2; NPV, negative predictive value; PPV, positive predictive value; PA, peanut allergic; PS, peanut sensitized but tolerant; NA, non-peanut-sensitised non-allergic; BAT, basophil activation test; NR, non-responders; OFC, oral food challenges.

**E-References**

1. Brough HA, Makinson K, Penagos M, Maleki SJ, Cheng H, Douiri A, et al. Distribution of peanut protein in the home environment. J Allergy Clin Immunol 2013.

2. Roberts G, Lack G. Diagnosing peanut allergy with skin prick and specific IgE testing. J Allergy Clin Immunol 2005; 115:1291-6.

3. Dang TD, Tang M, Choo S, Licciardi PV, Koplin JJ, Martin PE, et al. Increasing the accuracy of peanut allergy diagnosis by using Ara h 2. J Allergy Clin Immunol 2012; 129:1056-63.

4. Roberts G, Lack G. Food allergy--getting more out of your skin prick tests. Clin Exp Allergy 2000; 30:1495-8.

5. Vereda A, van Hage M, Ahlstedt S, Ibanez MD, Cuesta-Herranz J, van Odijk J, et al. Peanut allergy: Clinical and immunologic differences among patients from 3 different geographic regions. J Allergy Clin Immunol 2011; 127:603-7.

6. Nicolaou N, Poorafshar M, Murray C, Simpson A, Winell H, Kerry G, et al. Allergy or tolerance in children sensitized to peanut: prevalence and differentiation using component-resolved diagnostics. J Allergy Clin Immunol 2010; 125:191-7 e1-13.

7. Lieberman JA, Glaumann S, Batelson S, Borres MP, Sampson HA, Nilsson C. The utility of peanut components in the diagnosis of IgE-mediated peanut allergy among distinct populations. J Allergy Clin Immunol Pract 2013; 1:75-82.

8. Rubio A, Vivinus-Nebot M, Bourrier T, Saggio B, Albertini M, Bernard A. Benefit of the basophil activation test in deciding when to reintroduce cow's milk in allergic children. Allergy 2011; 66:92-100.

9. Wanich N, Nowak-Wegrzyn A, Sampson HA, Shreffler WG. Allergen-specific basophil suppression associated with clinical tolerance in patients with milk allergy. J Allergy Clin Immunol 2009; 123:789-94 e20.

10. Burks AW, Jones SM, Wood RA, Fleischer DM, Sicherer SH, Lindblad RW, et al. Oral immunotherapy for treatment of egg allergy in children. N Engl J Med 2012; 367:233-43.

11. Jones SM, Pons L, Roberts JL, Scurlock AM, Perry TT, Kulis M, et al. Clinical efficacy and immune regulation with peanut oral immunotherapy. J Allergy Clin Immunol 2009; 124:292-300.

12. Kim EH, Bird JA, Kulis M, Laubach S, Pons L, Shreffler W, et al. Sublingual immunotherapy for peanut allergy: clinical and immunologic evidence of desensitization. J Allergy Clin Immunol 2011; 127:640-6 e1.

13. Thyagarajan A, Jones SM, Calatroni A, Pons L, Kulis M, Woo CS, et al. Evidence of pathway-specific basophil anergy induced by peanut oral immunotherapy in peanut-allergic children. Clin Exp Allergy 2012; 42:1197-205.

14. Nopp A, Johansson SG, Ankerst J, Bylin G, Cardell LO, Gronneberg R, et al. Basophil allergen threshold sensitivity: a useful approach to anti-IgE treatment efficacy evaluation. Allergy 2006; 61:298-302.

15. Nopp A, Johansson SG, Ankerst J, Palmqvist M, Oman H. CD-sens and clinical changes during withdrawal of Xolair after 6 years of treatment. Allergy 2007; 62:1175-81.

16. Nopp A, Johansson SG, Adedoyin J, Ankerst J, Palmqvist M, Oman H. After 6 years with Xolair; a 3-year withdrawal follow-up. Allergy 2010; 65:56-60.

17. Sampson HA. Utility of food-specific IgE concentrations in predicting symptomatic food allergy. J Allergy Clin Immunol 2001; 107:891-6.
