# Supplementary material for: Basophil activation test discriminates between allergy and tolerance in peanut-sensitized children
Source: J Allergy Clin Immunol. 2014 Sep;134(3):645–52. doi: 10.1016/j.jaci.2014.04.039 (PMC4164910; doi:10.1016/j.jaci.2014.04.039)
Supplement: Tables E1–E8 [file mmc2.docx]

**Online Repository Material**

**E-Tables**

**Table E1 -** Doses of peanut protein in the oral food challenge protocol. Placebo doses were randomly interspersed with verum doses.

| **DOSES** | **Peanut protein (g)** |
| --- | --- |
| 1 | 0.1 |
| 2 | 0.25 |
| 3 | 0.5 |
| 4 | 1.0 |
| 5 | 2.5 |
| 6 | 5.0 |

**Table E2** – Criteria for positive oral food challenge to peanut. A positive oral food challenge (OFC) was defined by the presence of either ≥1 major criteria or ≥2 minor criteria. An indeterminate OFC was defined as one minor criterion. A negative OFC was defined by the absence of major or minor criteria.

| **Major criteria** | Confluent erythematous pruritic rash |
| --- | --- |
|  | Wheezing |
|  | Stridor |
|  | Dysphonia / Aphonia |
|  | ≥ 3 urticarial lesions |
|  | ≥ 1 site of angioedema |
|  | Hypotension for age not related to vasovagal episode |
|  | Evidence of severe abdominal pain that persists for ≥3 minutes |
| **Minor criteria** | Vomiting |
|  | Diarrhoea |
|  | Persistent rubbing of eyes that last ≥3 minutes |
|  | Persistent rhinorrhea that lasts ≥3 minutes |
|  | Persistent scratching that lasts ≥3 minutes |

**Table E3 -** Parameters of basophil activation test (BAT) in relation to allergy versus tolerance to peanut (n=92).

| **BAT to peanut** | **Peanut allergic**  **(n=42)** | **Peanut tolerant (n=50)** | | **p value*** | **AUC ROC curve**  **(95% CI)** |
| --- | --- | --- | --- | --- | --- |
|  |  | **PS (n=31)** | **NA (n=19)** |  |  |
| %CD63+ Peanut 0.1 | 0.83 (0.09, 7.18) | 0.02 (0, 0.20) | 0.13 (0, 0.39) | **<0.001** | 0.75 (0.65, 0.86) |
| %CD63+ Peanut 1 | 8.14 (1.57, 32.05) | 0 (0, 0.67) | 0.23 (0, 0.64) | **<0.001** | 0.92 (0.86, 0.98) |
| %CD63+ Peanut 10 | 35.88 (13.13, 60.71) | 0.13 (0, 0.77) | 0.25 (0.01, 0.58) | **<0.001** | 0.97 (0.94, 1.0) |
| %CD63+ Peanut 100 | 42.12 (19.96, 68.45) | 0.56 (0.11, 2.53) | 0.36 (0, 0.84) | **<0.001** | **0.99 (0.97, 1.0)** |
| %CD63+ Peanut 1,000 | 36.54 (23.46, 68.5) | 2.62 (0.16, 9.06) | 0.49 (0, 0.74) | **<0.001** | 0.96 (0.93, 1.0) |
| %CD63+ Peanut 10,000 | 47.42 (37.52, 67.79) | 5.24 (0.83, 19.83) | 0.41 (0.02, 0.80) | **<0.001** | 0.93 (0.87, 0.98) |
| SI CD203c Peanut 0.1 | 1.14 (1.0, 1.60) | 0.99 (0.93, 1.07) | 0.99 (0.91, 1.04) | **<0.001** | 0.75 (0.65, 0.85) |
| SI CD203c Peanut 1 | 1.56 (1.23, 3.03) | 0.98 (0.93, 1.05) | 1.0 (0.97, 1.03) | **<0.001** | 0.93 (0.87, 0.99) |
| SI CD203c Peanut 10 | 3.23 (2.11, 5.0) | 1.0 (0.96, 1.09) | 1.0 (0.96, 1.03) | **<0.001** | 0.98 (0.97, 1.0) |
| SI CD203c Peanut 100 | 4.27 (2.42, 5.36) | 1.08 (1.0, 1.44) | 1.0 (0.92, 1.08) | **<0.001** | **0.99 (0.98, 1.0)** |
| SI CD203c Peanut 1,000 | 4.0 (3.0, 6.6) | 1.18 (1.02, 1.67) | 0.97 (0.93, 1.02) | **<0.001** | 0.97 (0.93, 1.0) |
| SI CD203c Peanut 10,000 | 4.78 (3.46, 6.51) | 1.43 (1.1, 2.43) | 1.0 (0.97, 1.09) | **<0.001** | 0.93 (0.87, 0.98) |
| Mean CD63 Peanut 10-100 | 39.8 (19.7, 64.4) | 0.35 (0, 1.85) | 0.29 (0.4, 0.63) | **<0.001** | **0.99 (0.98, 1.0)** |
| Mean CD203c Peanut 10-100 | 3.69 (2.53, 5.09) | 1.04 (0.98, 1.29) | 1.0 (0.94, 1.05) | **<0.001** | **0.99 (0.99, 1.0)** |
| Mean CD63 Peanut 100-1,000 | 36.39 (25.52, 65.19) | 2.04 (0.34, 6.07) | 0.51 (0.10, 0.82) | **<0.001** | 0.98 (0.97, 1.0) |
| Mean CD203c Peanut 100-1,000 | 4.26 (2.79, 6.29) | 1.13 (1.05, 1.54) | 1.0 (0.95, 1.07) | **<0.001** | **0.99 (0.97, 1.0)** |
| AUC CD63 Peanut | 149.1 (88.6, 249.7) | 9.2 (1.5, 21.2) | 1.6 (0.5, 3.8) | **<0.001** | 0.98 (0.96, 1.0) |
| AUC CD203c Peanut | 16.9 (13.0, 22.8) | 5.5 (5.1, 6.6) | 5.0 (4.8, 5.3) | **<0.001** | **0.99 (0.97, 1.0)** |
| Maximal %CD63+ Peanut | 59.5 (39.5, 76.8) | 5.24 (1.6, 23.56) | 0.84 (0.42, 1.53) | **<0.001** | 0.95 (0.90, 0.99) |
| Maximal SI CD203c Peanut | 5.5 (4.0, 7.9) | 1.43 (1.2, 2.93) | 1.04 (1.0, 1.12) | **<0.001** | 0.95 (0.90, 0.99) |
| %CD63+ Peanut 100/aIgE | 1.14 (0.71, 1.54) | 0.02 (0.01, 0.13) | 0.02 (0, 0.11) | **<0.001** | 0.97 (0.95, 1.0) |
| %CD63+ Peanut 100/aFcɛRI | 1.92 (1.42, 4.49) | 0.03 (0.01, 0.21) | 0.02 (0, 0.11) | **<0.001** | 0.96 (0.91, 1.0)^$^ |
| EC50 (ng/ml) - CD63 | 10 (1, 10) | 300 (100, 1000)^#^ | - | **<0.001** | - |
| EC50 (ng/ml) - CD203c | 10 (1, 10) | 200 (100, 825)^+^ | - | **<0.001** | - |

Footnote: The largest areas under the ROC curves are highlighted in bold. Values are expressed as number (%) or median (inter-quartile range). *p value refers to the comparison between peanut allergic and PS. ^#^N=9 ^+^N=8 ^$^11 missing values where BAT to aFceRI was not performed.

Abbreviations: PS, peanut sensitized but tolerant; NA, non-peanut-sensitised non-allergic; AUC ROC, area under the receiver operator characteristic curve;,; CI, confidence interval; PE, peanut extract (the number refers to concentration in ng/ml - e.g. PE 10 means 10 ng/ml of peanut extract); ED, eliciting dose; EC50, half maximal effective concentration; SI, stimulation index; %CD63+ PE100/aIgE, ratio of %CD63+ basophils in PE 100 and anti-IgE (or aFcɛRI); fMLP, [Formyl-Methionyl-Leucyl-Phenylalanine](http://www.copewithcytokines.de/cope.cgi?key=Formyl%2dMethionyl%2dLeucyl%2dPhenylalanine).

**Table E4 -** Optimal cut-offs for the different parameters of basophil activation test to peanut (n=92).

| **BAT parameter** | **Cut-off (95% CI)** | **AUC ROC (95% CI)** | **Sensitivity (95%CI)** | **Specificity (95%CI)** | **PPV (95%CI)** | **NPV (95%CI)** | **LR+ (95%CI)** | **LR-(95%CI)** | **Diagnostic accuracy (95% CI)** |
| --- | --- | --- | --- | --- | --- | --- | --- | --- | --- |
| %CD63+ Peanut 0.1 | 0.39  (0.13; 2.94) | 0.73  (0.64; 0.82) | 64.3%  (48.0; 78.4) | 82.0%  (68.6; 91.4) | 75.0%  (57.8; 87.9) | 73.2%  (59.7; 84.2) | 3.6  (1.9; 6.7) | 0.44  (0.28; 0.67) | 73.9%  (64.9; 82.9) |
| %CD63+ Peanut 1 | 1.50  (0.56; 2.21) | 0.88  (0.81; 0.94) | 81.0%  (65.9; 91.4) | 94.0%  (83.5; 98.7) | 91.9%  (78.1; 98.3) | 85.5%  (73.3; 93.5) | 13.5  (4.5; 40.8) | 0.20  (0.11; 0.38) | 88.0%  (81.4; 94.7) |
| %CD63+ Peanut 10 | 2.33  (2.33; 7.04) | 0.96  (0.91; 1.0) | 95.2%  (83.8; 99.4) | 96.0%  (86.3; 99.5) | 95.2%  (83.8; 99.4) | 96.0%  (86.3; 99.5) | 23.8  (6.1; 92.7) | 0.05  (0.01; 0.19) | 95.7%  (91.5; 99.8) |
| %CD63+ Peanut 100 | 8.11  (2.93; 16.47) | 0.97  (0.93; 1.0) | 97.6%  (87.4; 99.9) | 96.0%  (86.3; 99.5) | 95.3%  (84.2; 99.4) | 98.0%  (89.1; 99.9) | 24.4  (6.3; 95.0) | 0.02  (0.0; 0.17) | 96.7%  (93.1; 100) |
| %CD63+ Peanut 1,000 | 20.31  (7.35; 21.71) | 0.92  (0.87; 0.98) | 90.5%  (77.4; 97.3) | 94.0%  (83.5; 98.7) | 92.7%  (80.1; 98.5) | 92.2%  (81.1; 97.8) | 15.1  (5.0; 45.4) | 0.10  (0.04; 0.26) | 92.4%  (87.0; 97.8) |
| %CD63+ Peanut 10,000 | 19.99  (9.60; 34.36) | 0.88  (0.82; 0.95) | 90.5%  (77.4; 97.3) | 86.0%  (73.3; 94.2) | 84.8%  (70.5; 93.5) | 91.5%  (79.6; 97.6) | 6.5  (3.2; 12.9) | 0.11  (0.04; 0.28) | 88.0%  (81.4; 94.7) |
| SI CD203c Peanut 0.1 | 1.10  (0.99; 1.30) | 0.72  (0.64; 0.81) | 54.8%  (38.7; 70.2) | 90.0%  (78.2; 96.7) | 82.1%  (63.1; 93.9) | 70.3%  (57.6; 81.1) | 5.5  (2.3; 13.1) | 0.50  (0.36; 0.71) | 73.9%  (64.9; 82.9) |
| SI CD203c Peanut 1 | 1.22  (1.06; 1.25) | 0.91  (0.85; 0.97) | 81.0%  (65.9; 91.4) | 100.0%  (92.9; 100) | 100.0%  (89.7; 100) | 86.2%  (74.6; 93.9) | -* | 0.19  (0.10; 0.36) | 91.3%  (85.5; 97.1) |
| SI CD203c Peanut 10 | 1.18  (1.18; 1.77) | 0.94  (0.89; 0.99) | 97.6%  (87.4; 99.9_) | 90.0%  (78.2; 96.7) | 89.1%  (76.4; 96.4) | 97.8%  (88.5; 99.9) | 9.8  (4.2; 22.5) | 0.03  (0.0; 0.18) | 93.5%  (88.4; 98.5) |
| SI CD203c Peanut 100 | 1.88  (1.62; 2.24) | 0.96  (0.91; 1.0) | 95.2%  (83.8; 99.4) | 96.0%  (86.3; 99.5) | 95.2%  (83.8; 99.4) | 96.0%  (86.3; 99.5) | 23.8  (6.1; 92.7) | 0.05  (0.01; 0.19) | 95.7%  (91.5; 99.8) |
| SI CD203c Peanut 1,000 | 1.96  (1.43; 2.62) | 0.93  (0.87; 0.98) | 95.2%  (83.8; 99.4) | 90.0%  (78.2; 96.7) | 88.9%  (75.9; 96.3) | 95.7%  (85.5; 99.5) | 9.5  (4.1; 21.9) | 0.05  (0.03; 0.21) | 92.4%  (87.0; 97.8) |
| SI CD203c Peanut 10,000 | 2.68  (1.95; 3.31) | 0.88  (0.82; 0.95) | 90.5%  (77.4; 97.3) | 86.0%  (73.3; 94.2) | 84.4%  (70.5; 93.5) | 91.5%  (79.6; 97.6) | 6.5  (3.2; 12.9) | 0.11  (0.04; 0.28) | 88.0%  (81.4; 94.7) |
| Mean CD63 Peanut 10-100 | 4.78  (4.78; 11.76) | 0.97  (0.93; 1.0) | 97.6%  (87.4; 99.9) | 96.0%  (86.3; 99.5) | 95.3%  (84.2; 99.4) | 98.0%  (89.1; 99.9) | 24.4  (6.3; 95.0) | 0.02  (0.0; 0.17) | 96.7%  (93.1; 100) |
| Mean CD203c Peanut 10-100 | 1.40  (1.40; 1.75) | 0.97  (0.94; 1.0) | 100%  (91.6; 100) | 94.0%  (83.5; 98.7) | 93.3%  (81.7; 98.6) | 100.0%  (92.5; 100) | 16.7  (5.6; 49.9) | -* | 96.7 %  (93.1; 100) |
| Mean CD63 Peanut 100-1,000 | 19.12  (5.14; 23.29) | 0.93  (0.88; 0.99) | 90.5%  (77.4; 97.3) | 96.0%  (86.3; 99.5) | 95.0%  (83.1; 99.4) | 92.3%  (81.5; 97.9) | 22.6  (5.8; 88.3) | 0.10  (0.04; 0.25) | 93.5%  (88.4; 98.5) |
| Mean CD203c Peanut 100-1,000 | 1.72  (1.72; 2.76) | 0.95  (0.91; 0.99) | 100%  (91.6; 100) | 90.0%  (78.2; 96.7) | 89.4%  (76.9; 96.5) | 100.0%  (92.1; 100) | 10.0  (4.4; 23.0) | -* | 94.6%  (89.9; 99.2) |
| AUC CD63 Peanut | 30.01  (24.91; 84.58) | 0.92  (0.87; 0.98) | 92.9%  (80.5; 98.5) | 92.0%  (80.8; 97.8) | 90.7%  (77.9; 97.4) | 93.9%  (83.1; 98.7) | 11.6  (4.5; 29.8) | 0.08  (0.03; 0.23) | 92.4%  (87.0; 97.8) |
| AUC CD203c Peanut | 8.41  (7.31; 10.67) | 0.95  (0.90; 0.99) | 97.6%  (87.4; 99.9) | 92.0%  (80.8; 97.8) | 91.1%  (78.8; 97.5) | 97.9%  (88.7; 99.9) | 12.2  (4.76; 31.3) | 0.03  (0; 0.18) | 94.6%  (89.9; 99.2) |
| Maximal %CD63+ Peanut | 27.52  (11.22; 34.66) | 0.90  (0.84; 0.96) | 92.9%  (80.5; 98.5) | 88.0%  (75.7; 95.5) | 86.7%  (73.2; 94.9) | 93.6%  (82.5; 98.7) | 7.7  (3.6; 16.5) | 0.08  (0.03; 0.24) | 90.2%  (84.1; 96.3) |
| Maximal SI CD203c Peanut | 2.65  (2.21; 3.76) | 0.90  (0.84; 0.96) | 97.6%  (87.4; 99.9) | 82.0%  (68.6; 91.4) | 82.0%  (68.6; 91.4) | 97.6%  (87.4; 99.9) | 5.42  (3.0; 9.8) | 0.03  (0.0; 0.20) | 89.1%  (82.8; 95.5) |
| %CD63+ Peanut 100/aIgE | 0.52  (0.12; 0.62) | 0.92  (0.86; 0.98) | 88.1%  (74.4; 96.0) | 96.0%  (86.3; 99.5) | 94.9%  (82.7; 99.4) | 90.6%  (79.3; 96.9) | 22.0  (5.6; 86.0) | 0.12  (0.05; 0.28) | 92.4%  (87.0; 97.8) |
| %CD63+ Peanut 100/aFcɛRI | 0.37  (0.37; 1.32) | 0.94  (0.89; 0.99) | 97.2%  (85.5; 99.9) | 91.1%  (78.8; 97.5) | 89.7%  (75.8; 97.1) | 97.6%  (87.4; 99.9) | 10.9  (4.3; 27.9) | 0.03  (0.0; 0.21) | 93.8%  (88.6; 99.1) |

Footnote: *LR could not be determined because sensitivity or specificity was 100%.

**Table E5** - Performance of different tests in the diagnosis of peanut allergy in the whole primary study population (n=92) and in the clinically equivocal population (n=39), excluding non-responders for whom there was no outcome of BAT.

| **Population** | **Diagnostic tests** | **Optimal cut-off** | **AUC ROC**  **(95% CI)** | **Sensitivity (95% CI)** | **Specificity (95% CI)** | **PPV (95% CI)** | **NPV**  **(95% CI)** | **LR+ (95% CI)** | **LR- (95% CI)** | **Diagnostic accuracy (95% CI)** |
| --- | --- | --- | --- | --- | --- | --- | --- | --- | --- | --- |
| Whole population (n=92) | SPT (mm) | 5  (5; 7) | 0.93  (0.88; 0.99) | 92.9%  (80.5; 98.5) | 94.0%  (83.5; 98.7) | 92.9%  (80.5; 98.5) | 94.0%  (83.5; 98.7) | 15.5  (5.15; 46.5) | 0.08  (0.03; 0.23) | 93.5%  (88.4; 98.5) |
|  | Specific IgE (KU/L) | 5.35  (1.04; 10.9) | 0.81  (0.73; 0.89) | 69.0%  (52.9; 82.4) | 92.0%  (80.8; 97.8) | 87.9%  (71.8; 96.6) | 78.0%  (65.3; 87.7) | 8.63  (3.3; 22.6) | 0.34  (0.21; 0.53) | 81.5%  (73.6; 89.5) |
|  | Ara h 2 (KU/L) | 0.53  (0.10; 2.29) | 0.91  (0.85; 0.97) | 83.3%  (68.6; 93.0) | 98.0%  (89.4; 99.9) | 97.2%  (85.5; 99.9) | 87.5 %  (75.9; 94.8) | 41.7  (5.96; 291) | 0.17  (0.09; 0.34) | 91.3%  (85.5; 97.1) |
|  | BAT (%CD63^10-100^) | 4.78  (4.78; 11.76) | 0.97  (0.93; 1.0) | 97.6%  (87.4; 99.9) | 96.0%  (86.3; 99.5) | 95.3%  (84.2; 99.4) | 98.0%  (89.1; 99.9) | 24.4  (6.3; 95.0) | 0.02  (0.0; 0.17) | 96.7%  (93.1; 100) |
| Sub- group with equivocal history, SPT and specific IgE results (n=39) | SPT (mm) | 7  (2; 7) | 0.80  (0.61; 0.98) | 62.5%  (24.5; 91.5) | 96.8%  (83.3; 99.9) | 83.3%  (35.9; 99.6) | 90.9%  (75.7; 98.1) | 19.4  (2.6; 143.0) | 0.39  (0.16; 0.95) | 89.7%  (80.2; 99.3) |
|  | Specific IgE (KU/L) | 0.25  (0.19; 14.5) | 0.46  (0.28; 0.63) | 75.0  (34.9; 96.8) | 16.1%  (5.5; 33.7) | 18.8%  (7.2; 36.4) | 71.4 %  (29.0; 96.3) | 0.9  (0.6; 1.4) | 1.55  (0.37; 6.57) | 28.2%  (14.1; 42.3) |
|  | Ara h 2 (KU/L) | 0.05  (0.05; 5.05) | 0.69  (0.61; 0.78) | 100.0  (63.1; 100) | 38.7%  (21.8; 57.8) | 29.6%  (13.8; 50.2) | 100%  (73.5; 100) | 1.6  (1.2; 2.2) | -* | 51.3%  (35.6; 67.0) |
|  | BAT (%CD63^10-100^) | 11.75  (1.43; 17.63) | 0.92  (0.80; 1.0) | 87.5  (47.3; 99.7) | 96.8 %  (83.3; 99.9) | 87.5%  (47.3; 99.7) | 96.8 %  (83.3; 99.9) | 27.1  (3.9; 190.0) | 0.13  (0.02; 0.81) | 95.0%  (87.9; 100) |

Abbreviations: AUC ROC, area under the receiver operator characteristic curve; S, sensitivity; Sp, specificity; PPV, positive predictive value; NPV, negative predictive value; SPT, skin prick test to peanut; Specific IgE, peanut-specific IgE; Ara h 2, specific IgE to Ara h 2; BAT, basophil activation test; CD63^10-100^, average percentage of CD63-positive basophils at 10 and 100ng/ml of peanut extract *LR could not be determined because sensitivity or specificity was 100%.

**Table E6** - Demographic and clinical characteristics of the external validation population (n=65).

|  | **Peanut allergic**  **(n=25)** | **Peanut tolerant**  **(n=40)** | | **p value*** |
| --- | --- | --- | --- | --- |
|  |  | **Peanut-sensitized but tolerant**  **(n=24)** | **Non-peanut sensitized non allergic**  **(n=16)** |  |
| Age (years) | 5.3 (1.7; 13.2) | 6.0 (0.5; 15.8) | 5.2 (4.8; 7.0) | 0.689 |
| Males - n (%) | 20 (80.0%) | 14 (58.3%) | 10 (62.5%) | 0.128 |
| History of oral exposure to peanut - n (%) | 8 (32.0%) | 8 (33.3%) | 9 (56.3%) | 1.0 |
| Skin prick test to peanut (mm) | 9 (1; 17) | 4 (0; 9) | 0 (0; 0) | **<0.001** |
| Specific IgE to peanut (KU_A_/L) | 6.13 (0.15; 194.0) | 1.53 (0.07; 22.20) | 0.01 (0.01; 0.08) | **0.021** |
| Specific IgE to Ara h 1 (KU_A_/L) | 0.21 (0.01; 74.70) | 0.08 (0.01; 11.70) | 0.01 (0.01; 0.05) | 0.128 |
| Specific IgE to Ara h 2 (KU_A_/L) | 1.65 (0.01; 142.0) | 0.12 (0; 7.0) | 0.01 (0.01; 0.01) | **<0.001** |
| Specific IgE to Ara h 3 (KU_A_/L) | 0.04 (0.01; 15.20) | 0.06 (0.01; 7.28) | 0.01 (0.01; 0.05) | 0.864 |
| Specific IgE to Ara h 8 (KU_A_/L) | 0.03 (0.01; 9.80) | 0.04 (0.01; 62.30) | 0.01 (0.01; 0.17) | 0.980 |
| Specific IgE to Ara h 9 (KU_A_/L) | 0.01 (0.01; 7.0) | 0.04 (0.01; 11.90) | 0.01 (0.01; 0.01) | 0.084 |
| Other food allergy - n (%) | 24 (96.0%) | 21 (87.5%) | 6 (37.5%) | 0.349 |
| Atopic eczema - n (%) | 20 (80.0%) | 18 (75.0%) | 14 (87.5%) | 0.742 |
| Asthma - n (%) | 14 (56.0%) | 6 (25.0%) | 7 (43.8%) | **0.042** |
| Allergic rhinitis - n (%) | 19 (76.0%) | 11 (45.8%) | 10 (62.5%) | **0.042** |
| Pollen allergy - n (%) | 12 (48.0%) | 11 (45.8%) | 7 (43.8%) | 1.0 |
| Non atopic - n (%) | 0 (0%) | 0 (0%) | 2 (12.5%) | - |

Footnote: Values are expressed as number (percentage) or median (range). *p value refers to the comparison between peanut allergic and peanut sensitized but tolerant patients (n=49). p values <0.05 are highlighted in bold.

**Table E7 -** External validation of diagnostic cut-offs of BAT in comparison with other allergy tests (n=61, non-responders were excluded). The following optimal cut-offs were applied: for BAT, CD63^10-100^ ≥4.78% ; for SPT, ≥5 mm; for peanut-specific IgE, ≥5.35 KU/L; and for Ara h 2-specific IgE, 0.53 KU/L.

| **Diagnostic tests** | **Sensitivity (95% CI)** | **Specificity (95% CI)** | **PPV**  **(95% CI)** | **NPV**  **(95% CI)** | **LR+ (95% CI)** | **LR-**  **(95% CI)** | **Diagnostic accuracy (95% CI)** |
| --- | --- | --- | --- | --- | --- | --- | --- |
| BAT | 83.3%  (74.0; 92.7) | 100.0%  (100.0; 100.0) | 100.0%  (100.0; 100.0) | 90.2%  (82.8; 97.7) | -* | 0.17  (0.07; 0.26) | 93.4%  (87.2; 99.7) |
| SPT | 83.3%  (74.0; 92.7) | 83.8%  (74.5; 93.0) | 76.9%  (66.3; 87.5) | 88.6%  (80.6; 96.6) | 5.14  (2.91; 13.31) | 0.20  (0.1; 0.3) | 83.6%  (74.3; 92.9) |
| Specific IgE | 50.0%  (37.5; 62.5) | 83.8%  (74.5; 93.0) | 66.7%  (54.8; 78.5) | 72.1%  (60.8; 83.3) | 3.08  (1.47; 8.98) | 0.60  (0.5; 0.7) | 70.5%  (59.0; 81.9) |
| Ara h 2 | 65.0%  (53.0; 77.0) | 97.2%  (93.1; 100.0) | 92.9%  (86.4; 99.3) | 83.3%  (74.0; 92.7) | 23.4  (7.68; -*) | 0.36  (0.2; 0.5) | 85.7%  (76.9; 94.5) |

Abbreviations: BAT, basophil activation test; SPT, skin prick test; Ara h 2, specifici IgE to Ara h 2; 95% CI, 95% confidence interval; PPV, positive predictive value; NPV, negative predictive value; LR+, positive likelihood ratio; LR-, negative likelihood ratio; *LR+ could not be determined as specificity was 100%.

**Table E8 -** Performance of Ara h 2-specific IgE as a second step in the diagnostic process, following SPT or specific IgE to peanut (N=104).

| **Ara h 2-specific IgE as a second step in the diagnostic process** | **Correct diagnoses*** | **False positives** | **False negatives** | **Change in number of OFC**** |
| --- | --- | --- | --- | --- |
| SPT → Ara h 2 | 93 (89%) | 2 (2%) | 3 (3%) | -30 (-83%) |
| Specific IgE → Ara h 2 | 84 (81%) | 3 (3%) | 4 (4%) | -23 (-64%) |

Footnote: Results are presented as number of patients (% of total study population). *The proportion of correct diagnoses was determined as ("true-positives"+"true-negatives")/n=104. **Reduction in OFC was calculated in comparison with the number of OFC following SPT and specific IgE (i.e. 36 OFC); negative numbers represent a decrease in the number of OFC required.
