# Supplementary figures and images for: Basophil activation test discriminates between allergy and tolerance in peanut-sensitized children
Source: J Allergy Clin Immunol. 2014 Sep;134(3):645–52. doi: 10.1016/j.jaci.2014.04.039 (PMC4164910; doi:10.1016/j.jaci.2014.04.039)

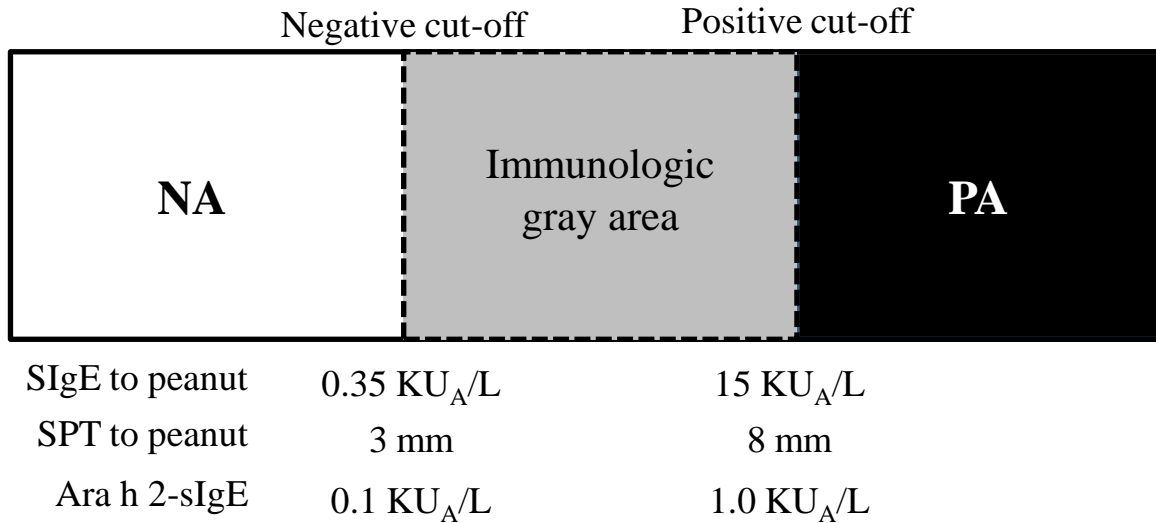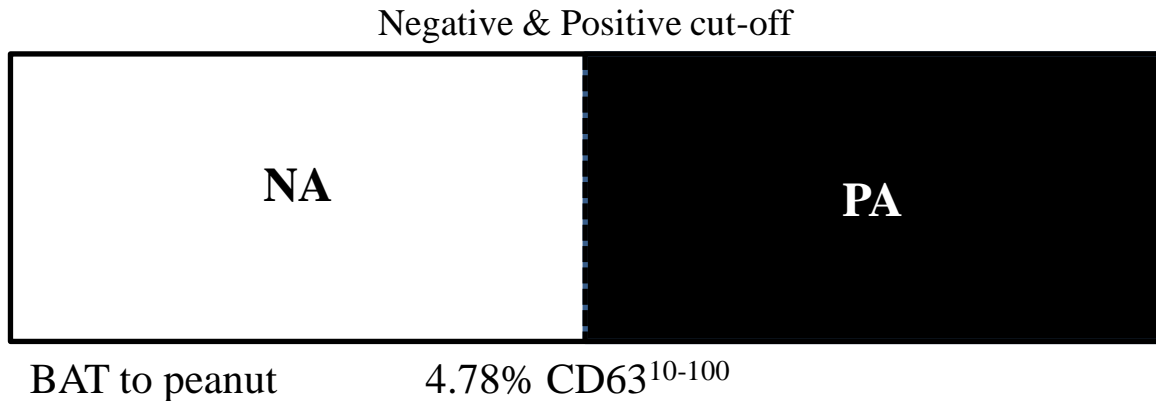

Supplement: Fig E1 [file mmc3.pdf]

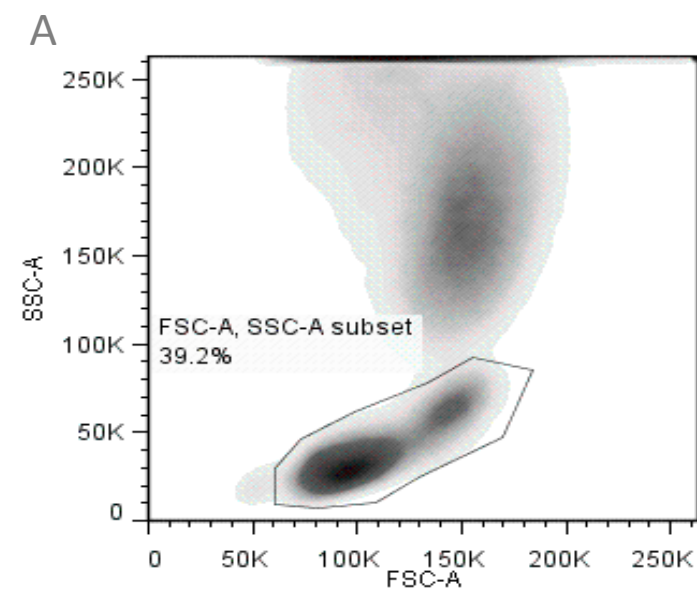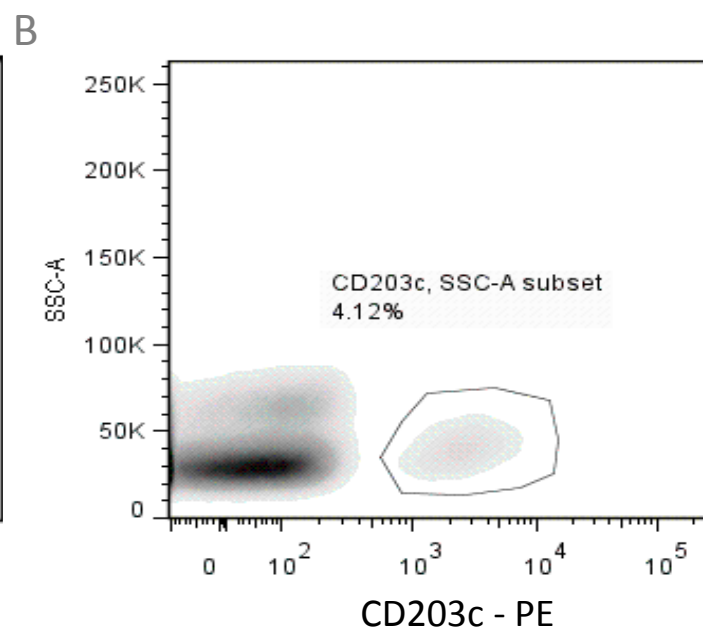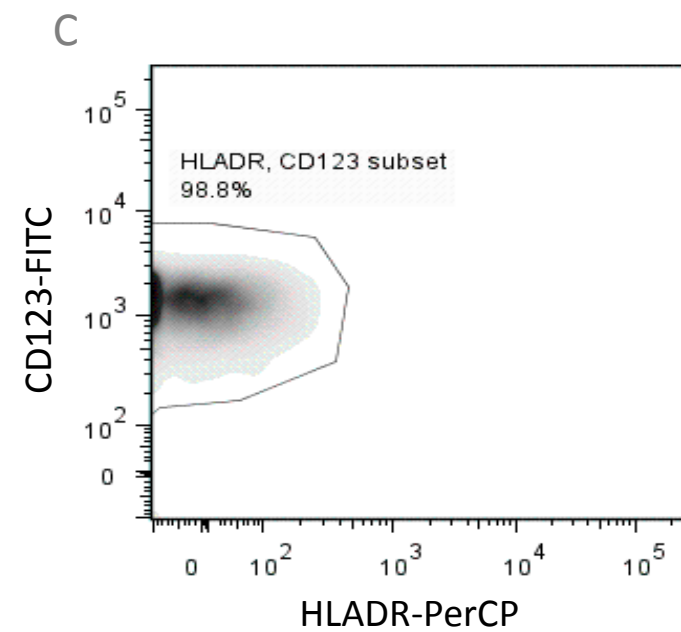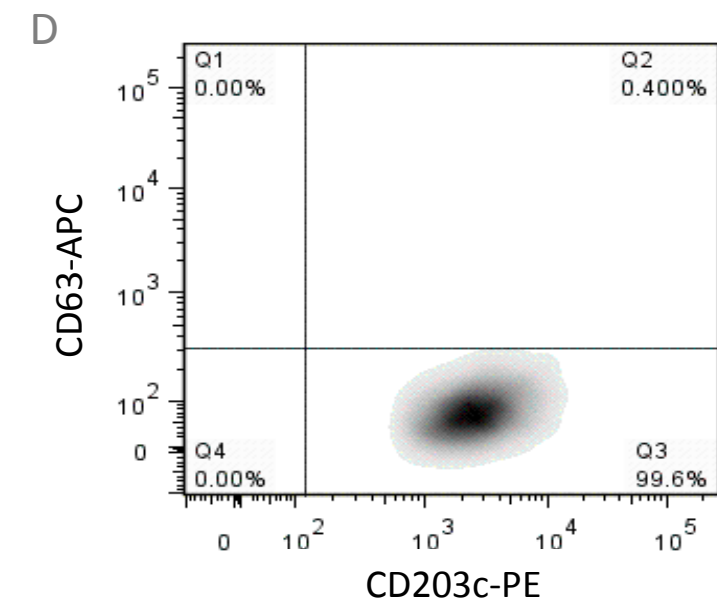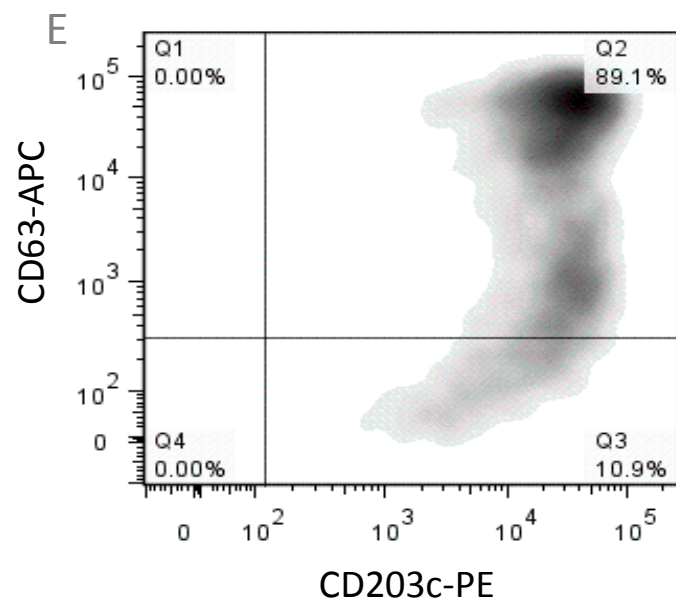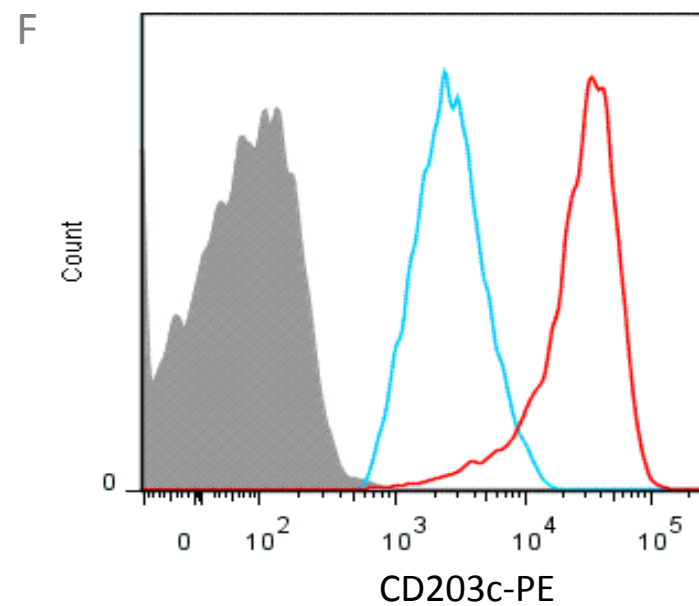

— Isotype control  
— Unstimulated basophils  
— Basophils stimulated with  
100ng/ml peanut extract

Supplement: Fig E2 [file mmc4.pdf]

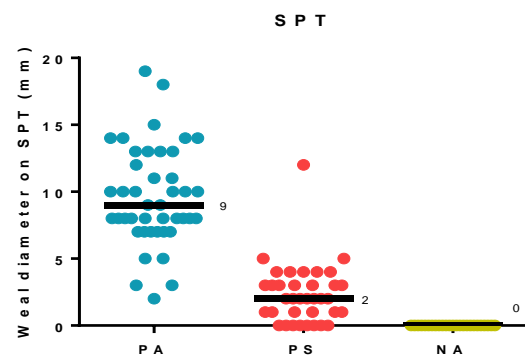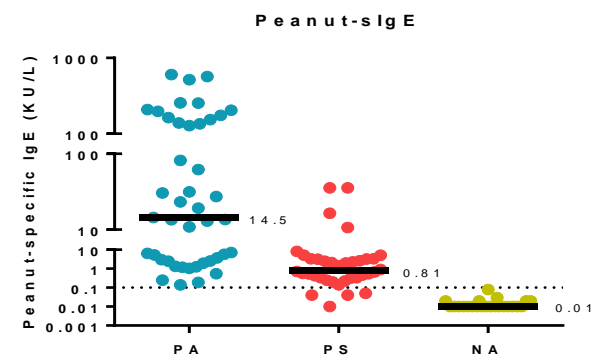

### Specific IgE to peanut components

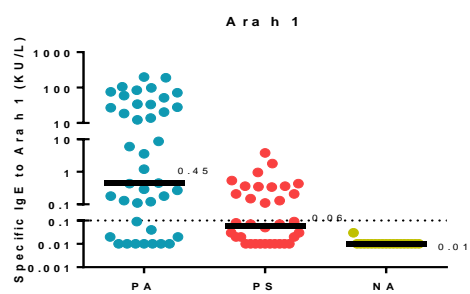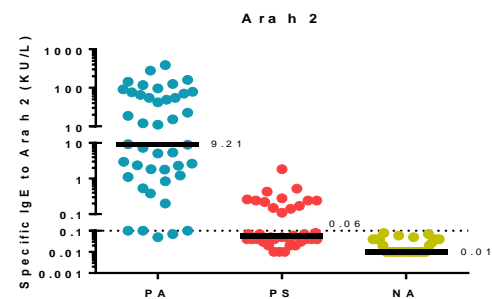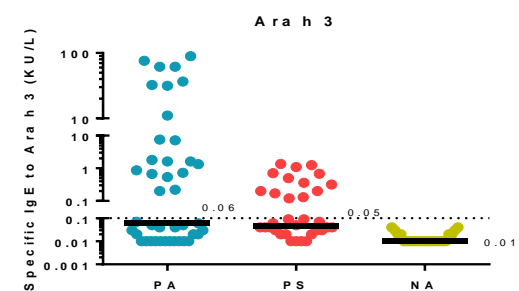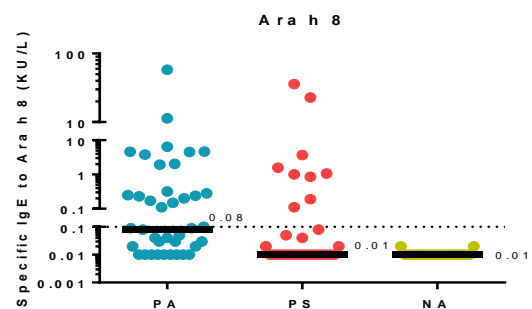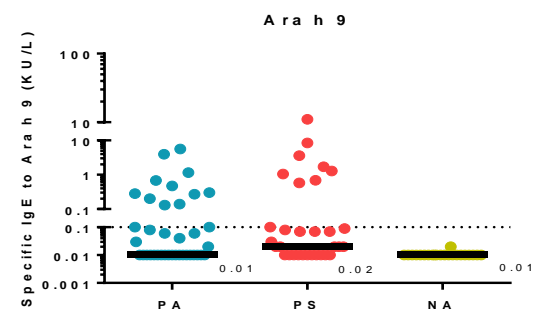

Supplement: Fig E5 [file mmc7.pdf]

Peanut allergic (n=42)

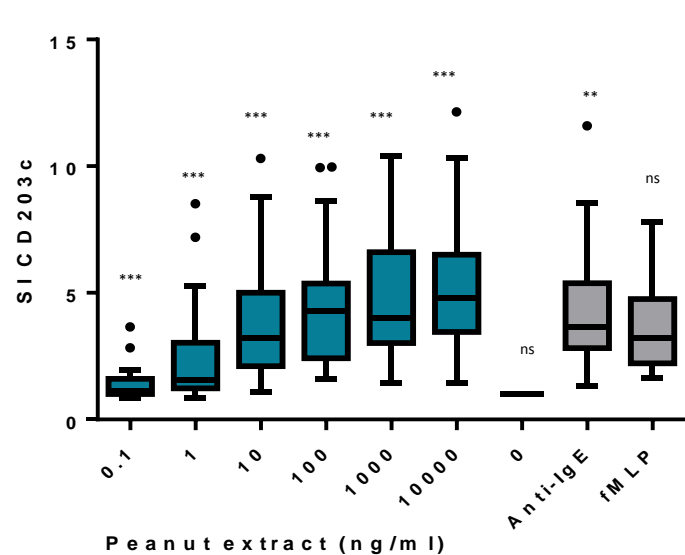

Peanut sensitised tolerant (n=31)

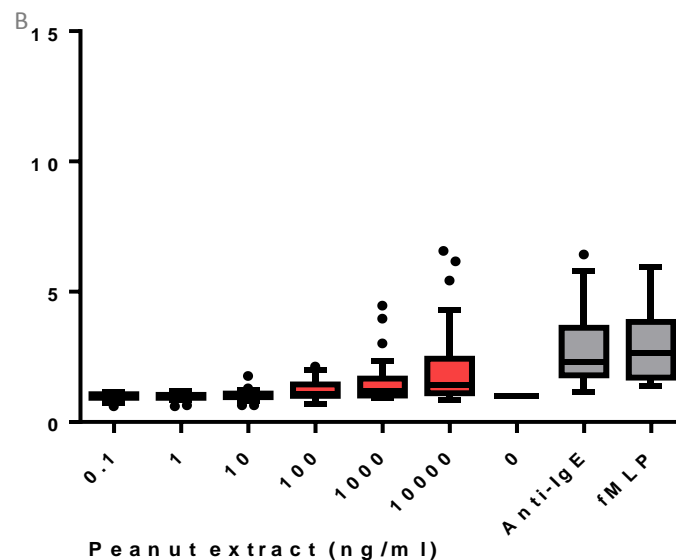

Non sensitised non allergic (n=19)

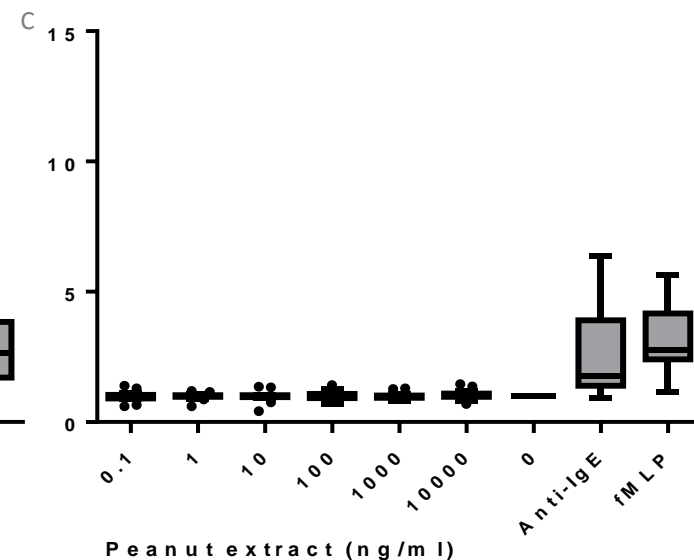

Supplement: Fig E6 [file mmc8.pdf]

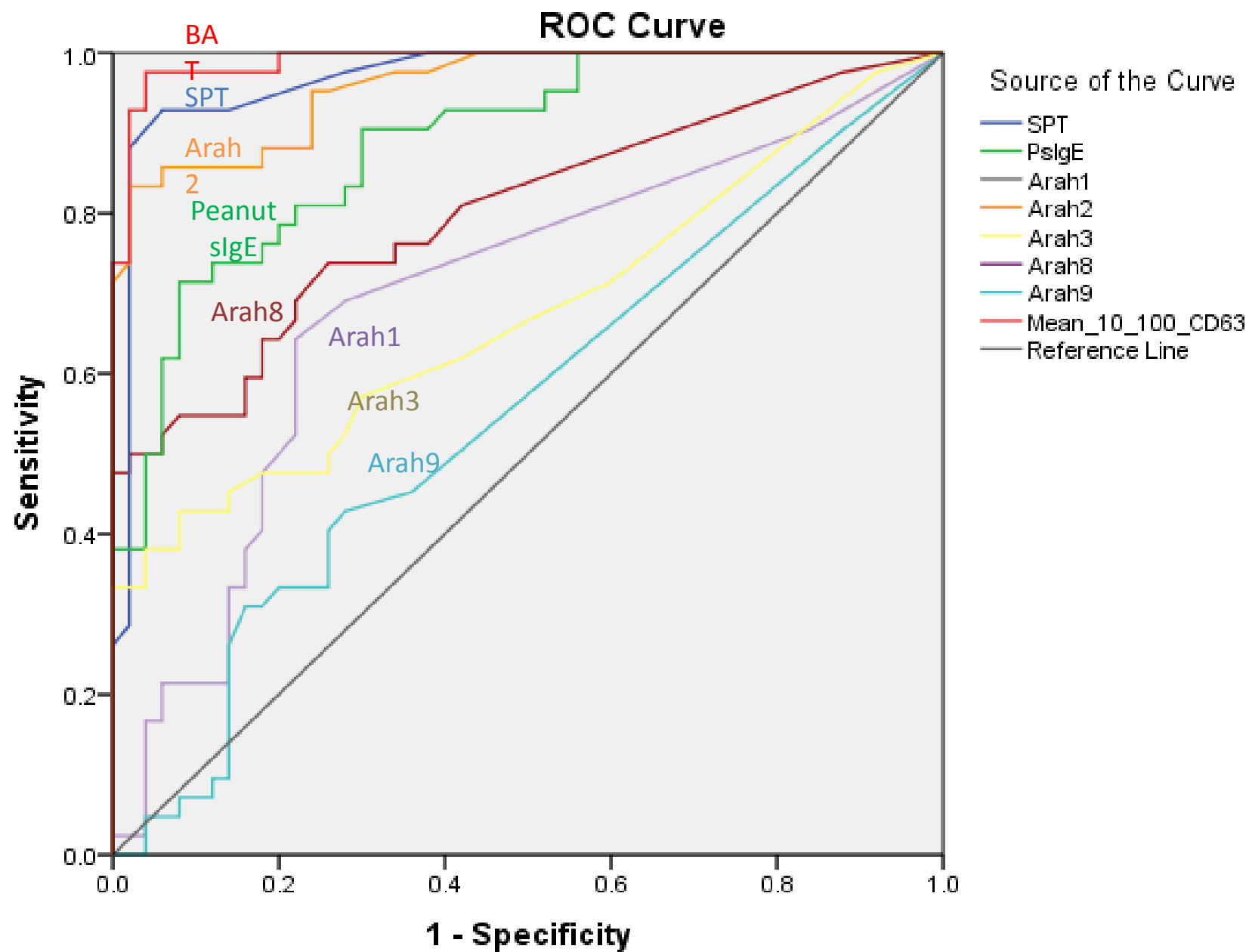

Diagonal segments are produced by ties.

Supplement: Fig E7 [file mmc9.pdf]

**A**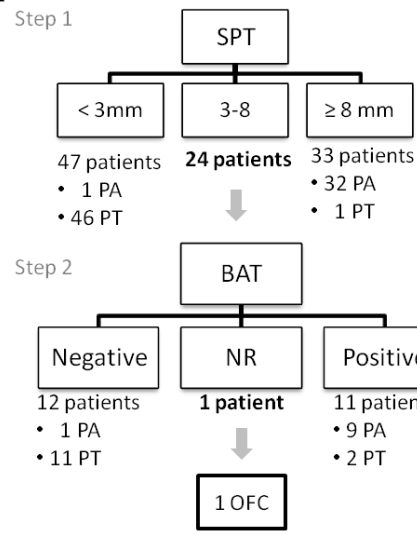**B**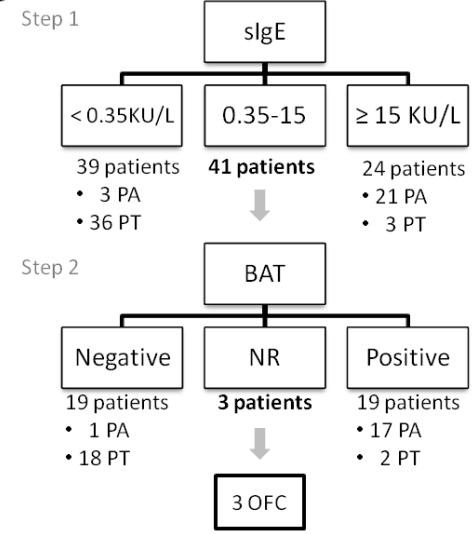**C**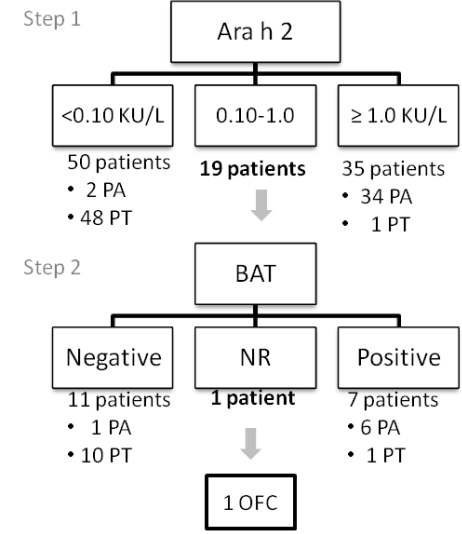**D**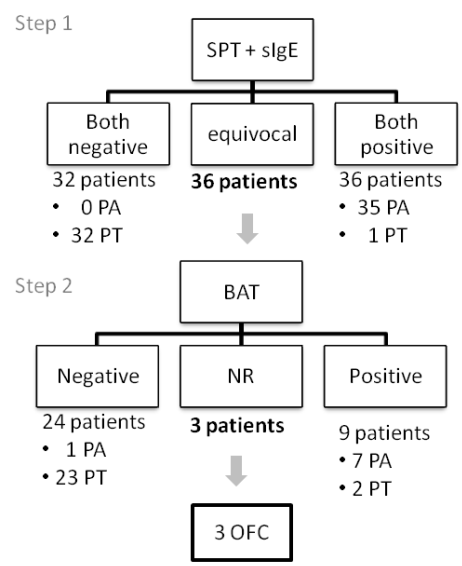**E**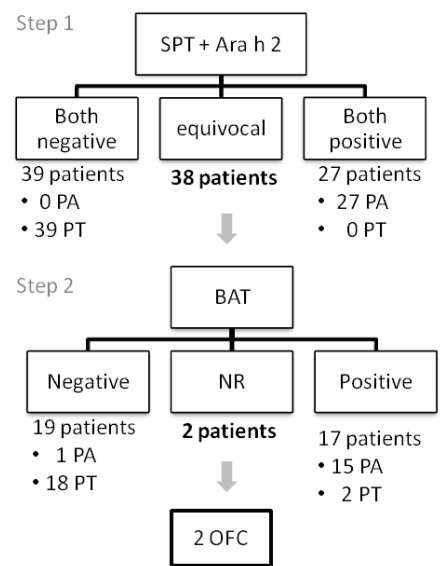**F**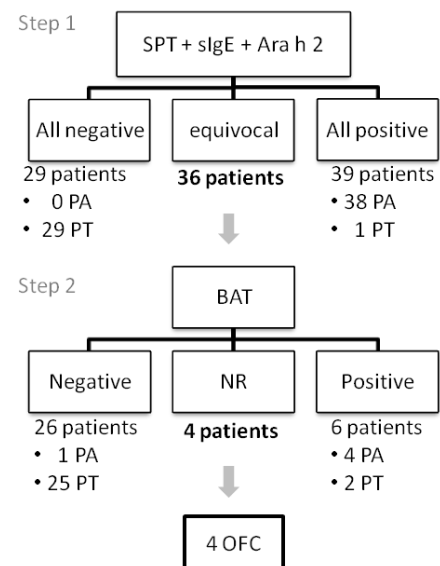**G**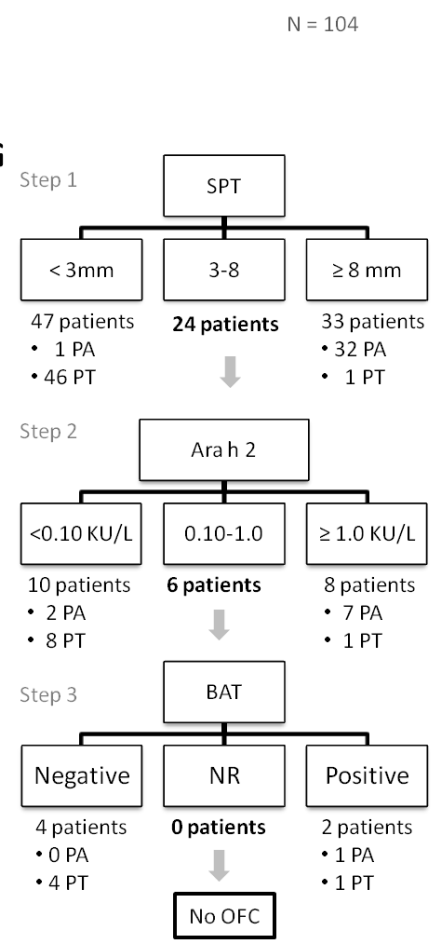

Supplement: Fig E8 [file mmc10.pdf]
