# Supplementary material for: Basophil activation test discriminates between allergy and tolerance in peanut-sensitized children
Source: J Allergy Clin Immunol. 2014 Sep;134(3):645–52. doi: 10.1016/j.jaci.2014.04.039 (PMC4164910; doi:10.1016/j.jaci.2014.04.039)
Supplement: Fig E3 [file mmc5.pdf]

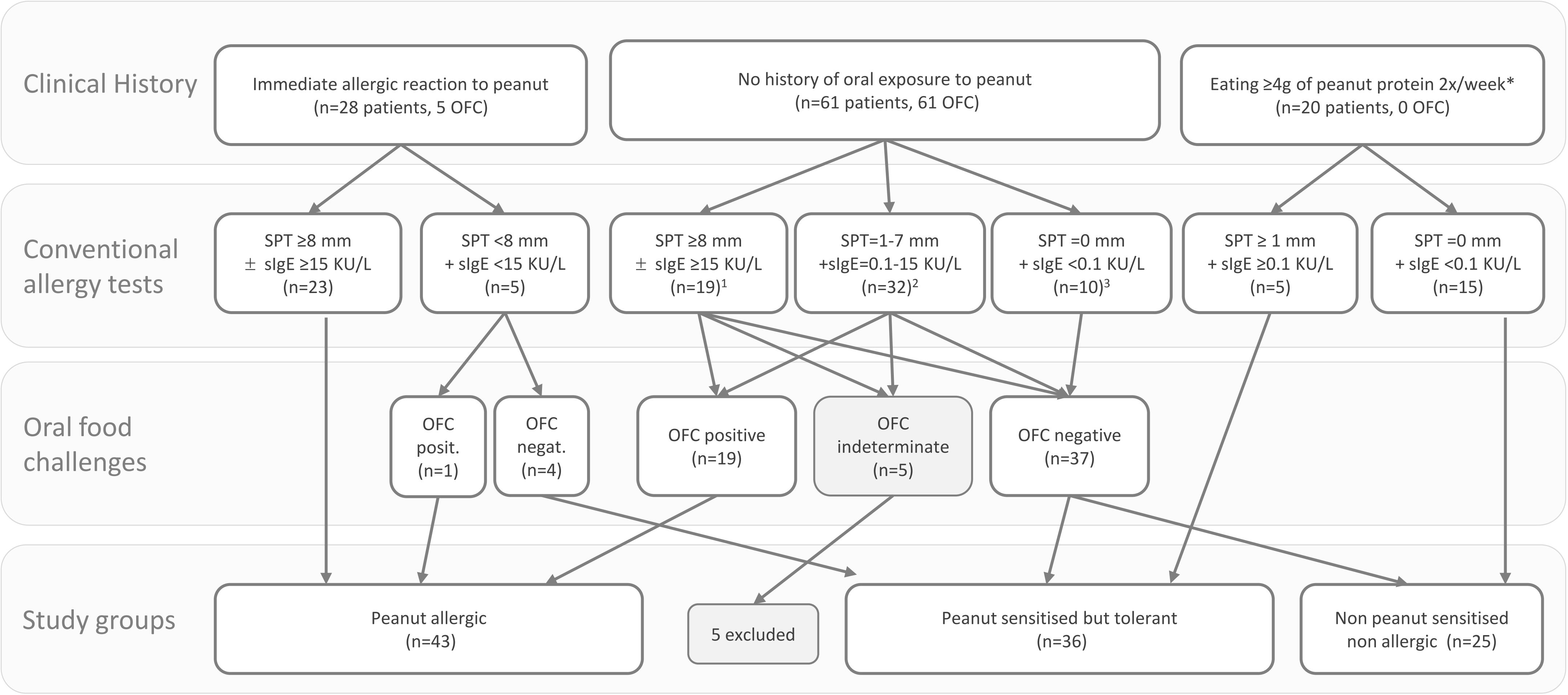

Clinical History
Immediate allergic reaction to peanut (n=28 patients, 5 OFC)
No history of oral exposure to peanut (n=61 patients, 61 OFC)
Eating ≥4g of peanut protein 2x/week* (n=20 patients, 0 OFC)
Conventional allergy tests
SPT ≥8 mm ± sIgE ≥15 KU/L (n=23)
SPT <8 mm + sIgE <15 KU/L (n=5)
SPT ≥8 mm ± sIgE ≥15 KU/L (n=19)1
SPT=1-7 mm +sIgE=0.1-15 KU/L (n=32)2
SPT =0 mm + sIgE <0.1 KU/L (n=10)3
SPT ≥ 1 mm + sIgE ≥0.1 KU/L (n=5)
SPT =0 mm + sIgE <0.1 KU/L (n=15)
Oral food challenges
OFC posit. (n=1)
OFC negat. (n=4)
OFC positive (n=19)
OFC indeterminate (n=5)
OFC negative (n=37)
Study groups
Peanut allergic (n=43)
5 excluded
Peanut sensitised but tolerant (n=36)
Non peanut sensitised non allergic (n=25)
