# Supplementary material for: Basophil activation test discriminates between allergy and tolerance in peanut-sensitized children
Source: J Allergy Clin Immunol. 2014 Sep;134(3):645–52. doi: 10.1016/j.jaci.2014.04.039 (PMC4164910; doi:10.1016/j.jaci.2014.04.039)
Supplement: Fig E4 [file mmc6.pdf]

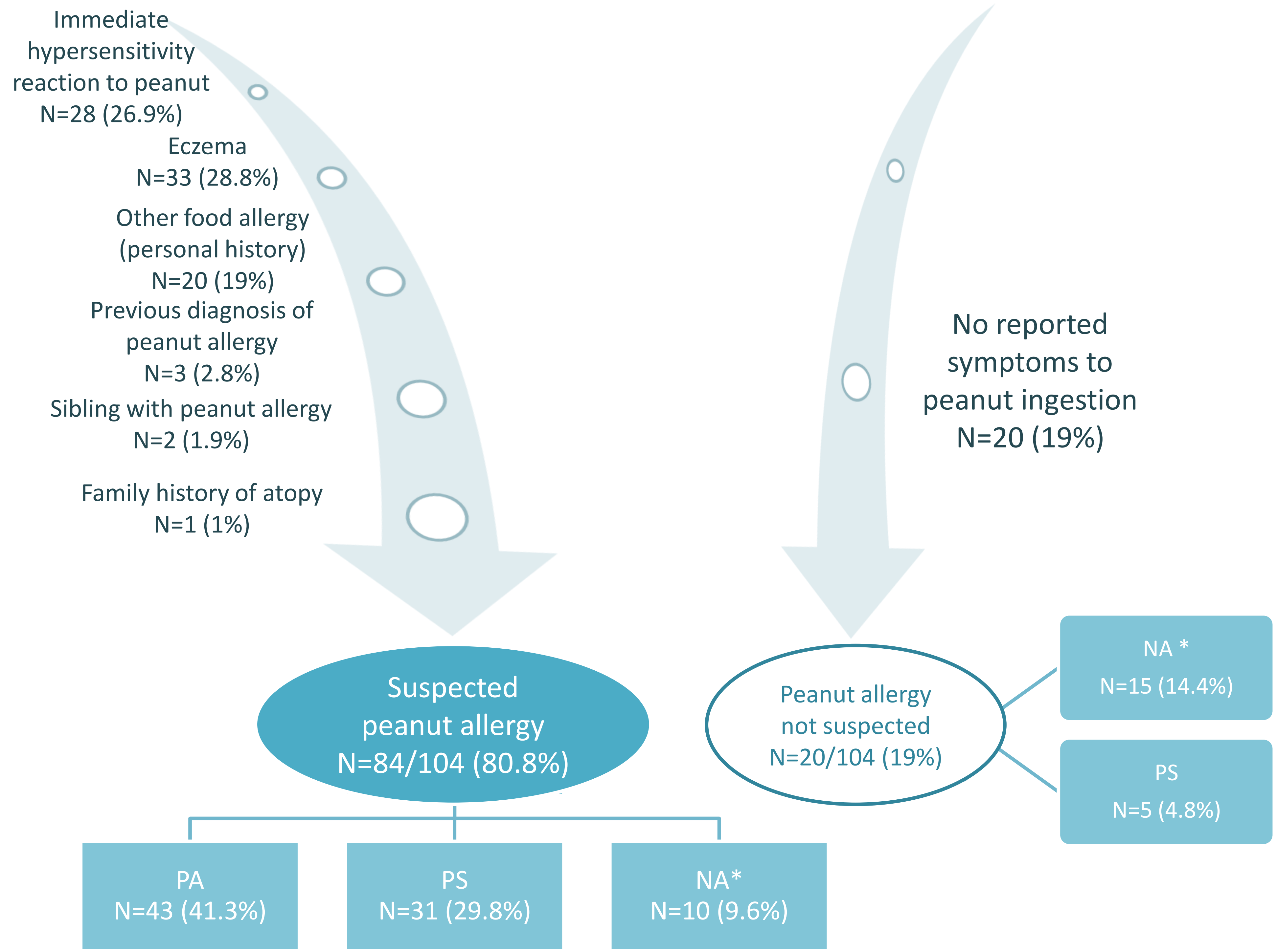

Immediate hypersensitivity reaction to peanut N=28 (26.9%)
Eczema N=33 (28.8%)
Other food allergy (personal history) N=20 (19%)
Previous diagnosis of peanut allergy N=3 (2.8%)
Sibling with peanut allergy N=2 (1.9%)
Family history of atopy N=1 (1%)
No reported symptoms to peanut ingestion N=20 (19%)
Suspected peanut allergy N=84/104 (80.8%)
Peanut allergy not suspected N=20/104 (19%)
NA * N=15 (14.4%)
PS N=5 (4.8%)
PA N=43 (41.3%)
PS N=31 (29.8%)
NA* N=10 (9.6%)
